# The SWI/SNF chromatin-remodeling subunit DPF2 facilitates NRF2-dependent antiinflammatory and antioxidant gene expression

**DOI:** 10.1172/JCI158419

**Published:** 2023-07-03

**Authors:** Gloria Mas, Na Man, Yuichiro Nakata, Concepcion Martinez-Caja, Daniel Karl, Felipe Beckedorff, Francesco Tamiro, Chuan Chen, Stephanie Duffort, Hidehiro Itonaga, Adnan K. Mookhtiar, Kranthi Kunkalla, Alfredo M. Valencia, Clayton K. Collings, Cigall Kadoch, Francisco Vega, Scott C. Kogan, Ramin Shiekhattar, Lluis Morey, Daniel Bilbao, Stephen D. Nimer

**Affiliations:** 1 Sylvester Comprehensive Cancer Center and; 2 Department of Human Genetics, University of Miami Miller School of Medicine, Miami, Florida, USA.; 3 Department of Pediatric Oncology, Dana-Farber Cancer Institute and Harvard Medical School, Boston, Massachusetts, USA.; 4 Broad Institute of MIT and Harvard, Cambridge, Massachusetts, USA.; 5 Chemical Biology Program, Harvard University, Cambridge, Massachusetts, USA.; 6 Department of Pathology and Laboratory Medicine, University of Miami Miller School of Medicine, Miami, Florida, USA.; 7 Helen Diller Family Comprehensive Cancer Center and; 8 Department of Laboratory Medicine, UCSF, San Francisco, California, USA.; 9 Department of Medicine, University of Miami Miller School of Medicine, Miami, Florida, USA.

**Keywords:** Hematology, Inflammation, Epigenetics, Hematopoietic stem cells, Macrophages

## Abstract

During emergency hematopoiesis, hematopoietic stem cells (HSCs) rapidly proliferate to produce myeloid and lymphoid effector cells, a response that is critical against infection or tissue injury. If unresolved, this process leads to sustained inflammation, which can cause life-threatening diseases and cancer. Here, we identify a role of double PHD fingers 2 (DPF2) in modulating inflammation. DPF2 is a defining subunit of the hematopoiesis-specific BAF (SWI/SNF) chromatin-remodeling complex, and it is mutated in multiple cancers and neurological disorders. We uncovered that hematopoiesis-specific *Dpf2*-KO mice developed leukopenia, severe anemia, and lethal systemic inflammation characterized by histiocytic and fibrotic tissue infiltration resembling a clinical hyperinflammatory state. *Dpf2* loss impaired the polarization of macrophages responsible for tissue repair, induced the unrestrained activation of Th cells, and generated an emergency-like state of HSC hyperproliferation and myeloid cell–biased differentiation. Mechanistically, *Dpf2* deficiency resulted in the loss of the BAF catalytic subunit BRG1 from nuclear factor erythroid 2-like 2–controlled (NRF2-controlled) enhancers, impairing the antioxidant and antiinflammatory transcriptional response needed to modulate inflammation. Finally, pharmacological reactivation of NRF2 suppressed the inflammation-mediated phenotypes and lethality of *Dpf2*^Δ/Δ^ mice. Our work establishes an essential role of the DPF2-BAF complex in licensing NRF2-dependent gene expression in HSCs and immune effector cells to prevent chronic inflammation.

## Introduction

In response to tissue injury, blood loss, or infection, hematopoietic stem cells (HSCs) exit quiescence, giving rise to highly proliferative progenitor cells, myeloid cells, and subtypes of T cells in a process known as emergency hematopoiesis. During this process, HSCs have increased cycling, myeloid-skewed differentiation, and sustained production of ROS (1). Emergency hematopoiesis is transient and occurs to maintain HSC fitness and survival and prevent the development of systemic inflammation and autoimmune diseases (2, 3). The mechanisms regulating emergency hematopoiesis and how its dysregulation contributes to chronic inflammatory conditions remain poorly understood.

BAF (SWI-SNF) complexes remodel chromatin and provide accessibility to transcription factors (TFs) to regulate gene expression. They contain a catalytic ATPase, either BRG1 or BRM, and additional subunits depending on the complex: the canonical BAF (cBAF) containing ARID1A/BAF250A/B and double PHD fingers 2 (DPF2)/BAF45D; the PBAF containing ARID2, PBRM1, BRD7, and PHF10/BAF45A; and the noncanonical BAF (ncBAF) containing BRD9 (4). While cBAF preferentially localizes to distal enhancers to regulate gene expression, ncBAF localizes to promoters and CCCTC-binding factor (CTCF) sites (5, 6). Mounting evidence indicates that subunits from all 3 complexes, including BAF53a, BAF45A, ARID1A, ARID2, BRM-SMARCA2, and BAF180 play roles in HSC function and the immune response (7–10). It is currently unknown whether BAF complexes also participate in the regulation of emergency hematopoiesis and prevent chronic inflammatory diseases.

Nuclear factor erythroid 2-like 2 (NRF2), encoded by NFE2L2, is a master TF that regulates HSC quiescence and antiinflammatory and antioxidant gene expression (11). NRF2 levels are regulated by kelch-like ECH-associated protein 1–mediated (KEAP1-mediated) proteasomal degradation (12). Upon inflammatory and oxidative stresses, NRF2 is released from KEAP1 and activates a potent cytoprotective response (13). NRF2 is expressed at relatively high levels in HSCs in the absence of stimuli (14) to maintain the basal expression of antioxidant and detoxification genes (15, 16). The inability of NRF2 to regulate its target genes in HSCs causes increased apoptosis, proliferation, and self-renewal as well as reduced homing and engraftment upon transplantation (17–21). Consequently, *Nrf2^–/–^* mice display thrombocytopenia and anemia, histiocytic infiltrations, and inflammatory lesions and are predisposed to autoimmune disease (11, 22, 23).

DPF2/BAF45D is a defining subunit of the cBAF complex in hematopoietic cells (24). Loss-of-function *Dpf2* mutations are found in cancer and in patients with Coffin-Siris syndrome (25, 26). Work from our laboratory and others has shown that DPF2 regulates myelopoiesis (27–29). DPF2 also interacts with NF-κB to control immune response genes in cancer cell lines (30–32). Here, we report that hematopoiesis-specific *Dpf2*-KO mice developed a lethal inflammatory disease involving dysfunctional HSCs, macrophages, and Th cells — phenotypes that mirror NRF2 deficiency. Mechanistically, NRF2 binding to active enhancers in HSCs depends on DPF2, and pharmacological reactivation of NRF2 overcomes the inflammatory defects driven by DPF2 loss, thereby prolonging survival. Our work uncovers the multilineage control of inflammation by DPF2, mediated by NRF2, establishing a role for the BAF complex in modulating inflammation.

## Results

### Hematopoiesis-specific loss of Dpf2 leads to systemic inflammation, reactive histiocytic infiltrations in multiple organs, and early death.

To examine the function of DPF2 in hematopoiesis, we generated hematopoiesis-specific *Mx1-Cre*– and *Vav1-Cre*–derived *Dpf2*^Δ/Δ^ mice. *Mx1-Cre*–driven expression of Cre recombinase is accomplished by administration of polyinosine-polycytidylic acid [poly(I:C)]. Unexpectedly, one-third of the *Mx1-Cre Dpf2^fl/fl^* mice died during or shortly after poly(I:C) administration (Supplemental Figure 1A; supplemental material available online with this article; https://doi.org/10.1172/JCI158419DS1). The surviving *Mx1-Cre Dpf2*^Δ/Δ^ mice had a shorter lifespan, residual *Dpf2* expression, and cytopenia (Supplemental Figure 1, A–C), suggesting that DPF2 may be essential for surviving inflammation triggered by poly(I:C). End-stage *Mx1-Cre Dpf2*^Δ/Δ^ mice displayed perivascular infiltrates in the liver and intra-alveolar infiltrates in the lungs, which were primarily histiocytic/myeloid cells, based on morphology and staining for the macrophage marker CD68 (Supplemental Figure 1, D and E). Histiocytic infiltrations are commonly found in aging mice and are associated with inflammatory and autoimmune disorders (33, 34). The infiltrates also stained positive for CD69, a marker of lymphocyte activation and therefore indicative of inflammation (Supplemental Figure 1F). Although the incomplete *Mx1-Cre*–mediated deletion of *Dpf2* precluded further analysis of this model, these data indicate that Dpf2 may be necessary for survival from acute inflammatory insults.

**Figure 1:**
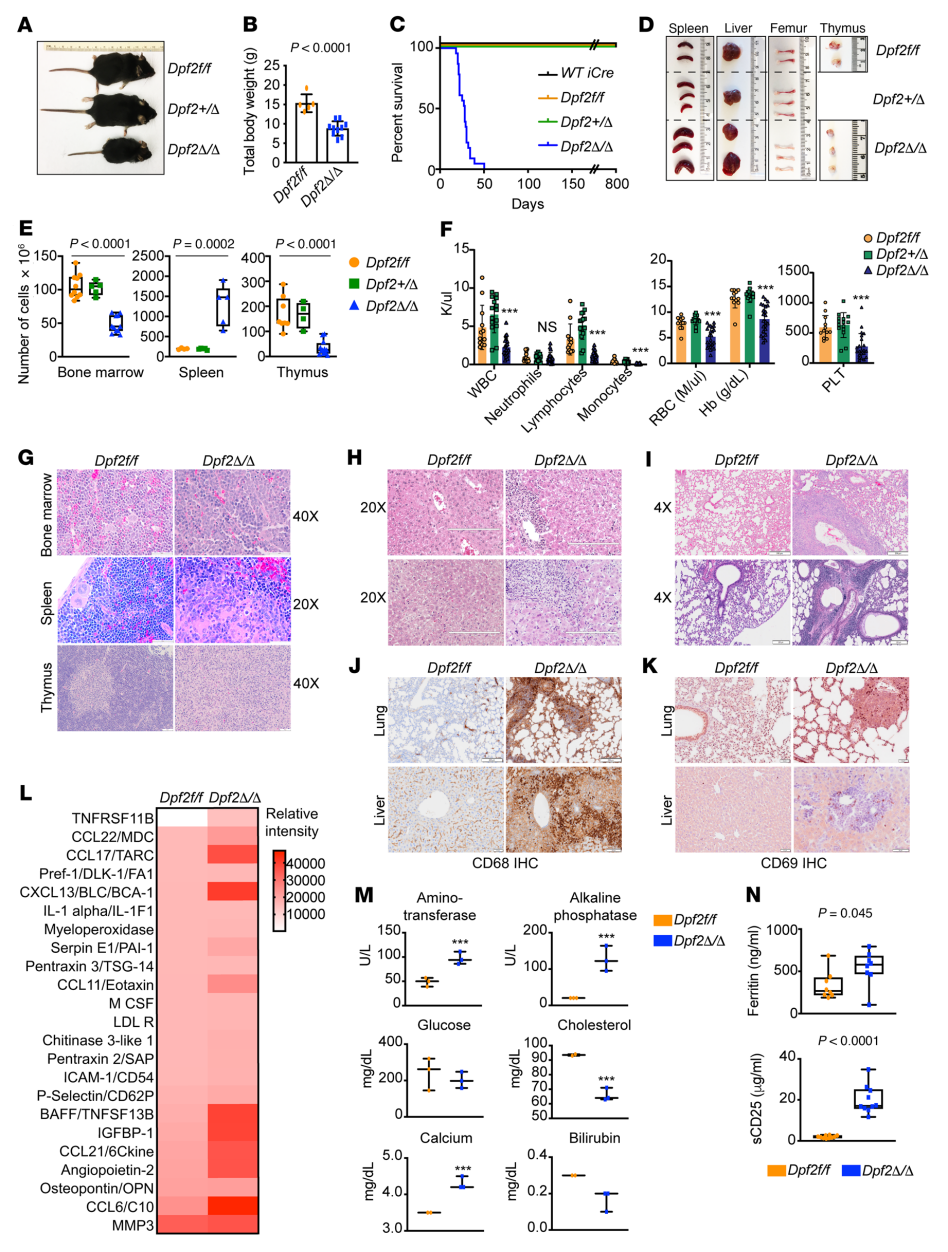
Hematopoiesis-specific, *Vav1-Cre*–mediated loss of *Dpf2* leads to premature death from pancytopenia and inflammatory lesions. (**A**) Representative images of *Vav1-Cre*–derived 28-day-old mice. (**B**) Total BWs of 28-day-old mice. (**C**) Kaplan-Meier survival curves; the median survival of *Dpf2*^Δ/Δ^ mice was 28 days. (**D**) Representative images of organs from 28-day-old mice. (**E**) Total number of cells in BM, spleen, and thymus. (**F**) CBC of PB in approximately 28-day-old mice (*n* = 18). Hb, hemoglobin; PLT, platelets. (**G**) Representative H&E staining of BM, spleen, and thymus from 28-day-old mice. Scale bars: 50 μm. (**H** and **I**) Representative H&E staining of liver (**G**) and lung (**H**) from end-stage *Dpf2*^Δ/Δ^ and age-matched *Dpf2^fl/fl^* mice. Scale bars: 200 μm. (**J**) Representative CD68/macrosialin IHC staining of lung and liver. Scale bars: 200 μm (lung IHC), 50 μm (*Dpf2^fl/fl^* liver), and 100 μm (*Dpf2*^Δ/Δ^ liver). (**K**) Representative CD69 IHC staining of lung and liver infiltrates. Scale bars: 50 μm. (**L**) Plasma cytokine levels. Values correspond to the mean spot pixel density relative to background from 4 mice/genotype. (**M**) Chemistry profiling of PB from 28-day-old mice (*n* = 3). (**N**) Serum ferritin and sCD25 plasma levels. All bar graph data represent the mean ± SD. ***P < 0.001, by 2-tailed, unpaired Student’s *t* test (**B**, **M**, and **N**) and ordinary, 1-way ANOVA (**E** and **F**).

The *Vav1-Cre*–derived *Dpf2*^Δ/Δ^ mice showed complete deletion of *Dpf2* in peripheral blood (PB) and bone marrow (BM) cells (Supplemental Figure 1G). *Dpf2*^Δ/Δ^ mice were born below the expected Mendelian ratio, suggesting partial embryonic lethality, and were smaller than the heterozygous or control mice (Figure 1, A and B, and Supplemental Figure 1H). The *Dpf2*^Δ/Δ^ mice survived a median of 28 days (Figure 1C) and had hepatosplenomegaly, pale BM, thymus atrophy, and increased spleen cellularity (Figure 1, D and E, and Supplemental Figure 1H). BM cellularity was 100%, with a left shift of the myeloid lineage (Figure 1G and Supplemental Figure 1I). End-stage *Dpf2*^Δ/Δ^ mice showed pancytopenia with lymphopenia and monocytopenia (Figure 1F). *Dpf2*^Δ/Δ^ PB smears showed polychromasia, anisocytosis, and Howell-Jolly bodies in RBCs (Supplemental Figure 1J). The splenic and thymic architecture was disrupted, with expanded splenic red pulp and near obliteration of white pulp (Figure 1G). Similar to *Mx1-Cre Dpf2*^Δ/Δ^ mice, *Vav1-Cre* *Dpf2*^Δ/Δ^ mice displayed prominent infiltrates in the BM, liver, and lungs, but not in the kidneys, heart, or brain (Figure 1, G–I). These infiltrates stained positive for the macrophage/histiocyte markers CD68 and galectin-3/MAC2 (35, 36) (Figure 1J and Supplemental Figure 1K) and CD69 (Figure 1K). End-stage *Dpf2*^Δ/Δ^ mice had elevated plasma levels of proinflammatory cytokines and chemokines, including TNFRSF11B, CCL22/MDC, CCL17/TARC, CXCL13/BLC, IL-1α, CCL11, and BAFF/TNFSF13B (Figure 1L). Many of these cytokines are secreted by DCs and macrophages (37–39). In addition, aminotransferase and alkaline phosphatase levels were elevated in plasma from *Dpf2*^Δ/Δ^ mice, reflecting liver damage (Figure 1M). *Dpf2*^Δ/Δ^ mice had high serum ferritin and sCD25 levels, clinical markers of macrophage activation syndrome (MAS) and hemophagocytic lymphohistiocytosis (HLH) (40), as well as reticulin fibrosis in BM and liver (Figure 1N and Supplemental Figure 1L). Many of the phenotypes of *Dpf2*^Δ/Δ^ mice resemble those of patients diagnosed with MAS and HLH, implicating the BAF complex in controlling inflammation.

### Dpf2^Δ/^ ^Δ^ macrophages are hyperproliferative and have impaired NRF2-target gene expression.

**Figure 2:**
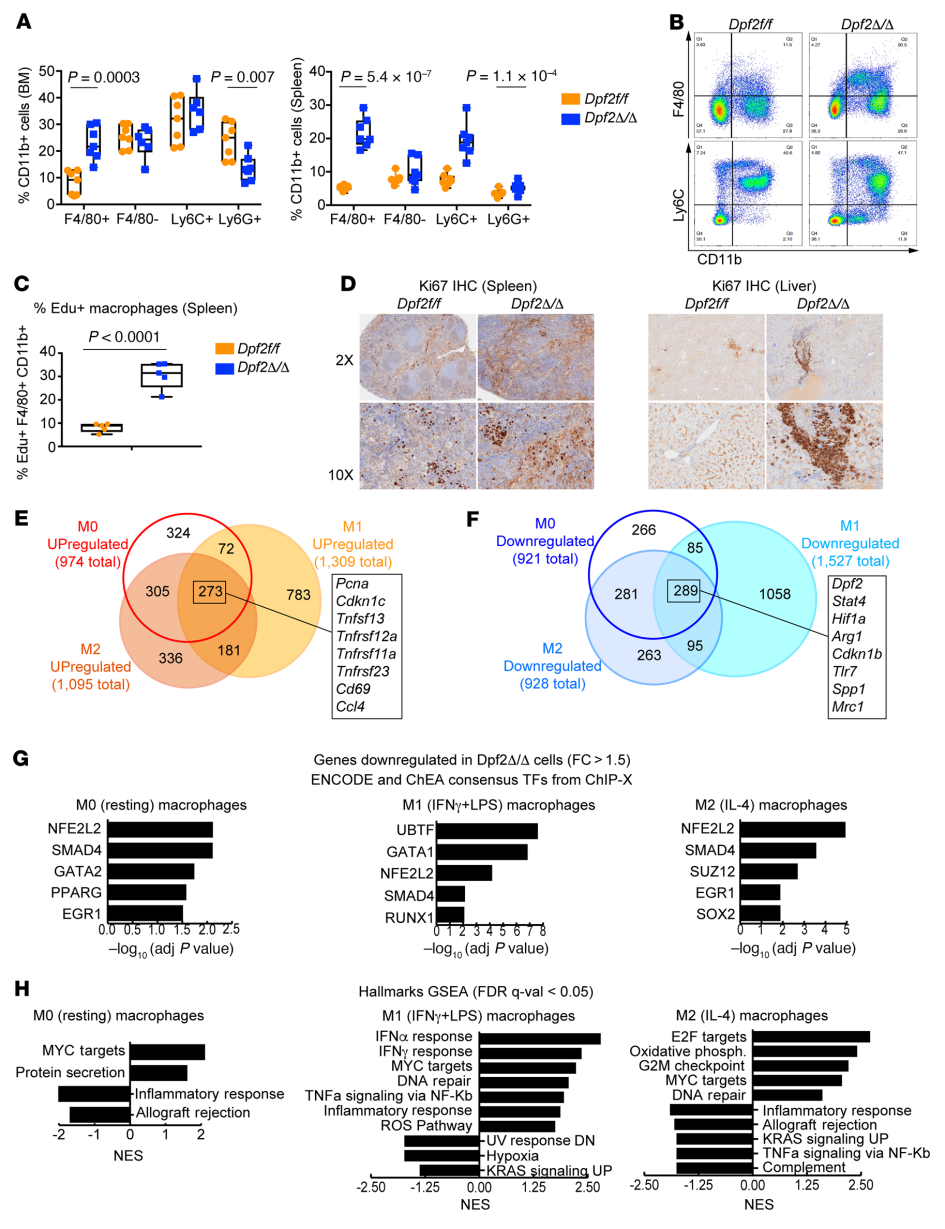
*Dpf2* loss results in hyperproliferation and infiltration of macrophages. (**A**) Frequency of myeloid cell populations (gated on CD11b^+^). (**B**) Representative FACS plot of BM myeloid cell populations. (**C**) Percentage of EdU^+^ splenic macrophages (F4/80^+^CD11b^+^) in end-stage *Dpf2*^Δ/Δ^ and age-matched *Dpf2^fl/fl^* mice. (**D**) Ki67 IHC staining of spleen and liver sections. Original magnification, ×2 and ×10. (**E**) Overlap between genes upregulated after Dpf2 loss in M0, M1, and M2 BMDMs (*q* < 0.05, fold change >1.5). Highlighted are a few of the 289 genes that were upregulated in all conditions. (**F**) Same as in **E**, but for genes that were downregulated. (**G**) ChEA of genes downregulated in *Dpf2*^Δ/Δ^ compared with *Dpf2^fl/fl^* BMDMs. (**H**) Hallmark GSEA of gene expression programs enriched in *Dpf2*^Δ/Δ^ BMDMs. NES, normalized enrichment score; DN, downregulated; UP, upregulated. Data represent the mean ± SD. *P* values were calculated using a 2-tailed, unpaired Student’s *t* test.

We analyzed *Dpf2*^Δ/Δ^ BM and splenic myeloid cell populations and found an increased frequency of CD11b^+^ myeloid cells, due to an increase in macrophages (CD11b^+^F4/80^+^) but not granulocytes (CD11b^+^Ly6G^+^) (Figure 2, A and B). Accumulation of BM macrophages with a reduction in PB monocytes (Figure 1F) could indicate an alteration in the location of F4/80^+^ cells and/or the proliferation and differentiation of *Dpf2*^Δ/Δ^ monocytes. In vivo EdU^+^ assays showed that *Dpf2*^Δ/Δ^ F4/80^+^ splenic macrophages were highly proliferative (Figure 2C), and the spleen, liver, and lung histiocytic infiltrates showed increased Ki67 staining (Figure 2D and Supplemental Figure 2A). BM-derived macrophages (BMDMs) from end-stage *Dpf2*^Δ/Δ^ and *Dpf2^fl/fl^* mice were morphologically similar, with a normal phagocytic capacity (Supplemental Figure 2, B and C), indicating that *Dpf2*-null macrophage precursors differentiated normally. We next polarized the macrophages with IFN-γ and LPS, or with IL-4, to generate classically or alternatively activated macrophages, respectively. While the polarization of *Dpf2*^Δ/Δ^ and *Dpf2^fl/fl^* cells to M1 macrophages (CD80^+^) was comparable, the polarization of M2 macrophages (CD206^+^) was severely impaired (Supplemental Figure 2D). Quantitative reverse transcription PCR (qRT-PCR) assays confirmed the reduced expression of classical M2 markers (*Mrc1/Cd206* and *Arg1)* and antiinflammatory and tissue repair markers (*Chil3* and *Fn1)* (Supplemental Figure 2E). Thus, DPF2 loss increased macrophage proliferation but impaired the function of inflammation-resolving, M2-type macrophages.

RNA-Seq of BMDMs (Supplemental Figure 2, F and G, and Supplemental Table 1) showed that the absence of DPF2 resulted in more than 900 differentially expressed genes under resting conditions (*q* < 0.05, fold change >1.5; Supplemental Figure 2H). Most genes deregulated in resting *Dpf2*^Δ/Δ^ macrophages were also altered in IL-4–stimulated *Dpf2*^Δ/Δ^ macrophages (Figure 2, E and F, and Supplemental Figure 2G), suggesting impaired activation of M2 macrophages. Regardless of the treatment condition, genes upregulated in *Dpf2*^Δ/Δ^ macrophages were enriched for E2F4 targets (Supplemental Figure 2I), a transcriptional repressor critical for cell quiescence (41). Moreover, cell-cycle and DNA replication pathways were enriched upon DPF2 loss (Supplemental Figure 2J), indicating that increased E2F4 target gene expression may be a mechanism underlying the hyperproliferation of *Dpf2*^Δ/Δ^ macrophages.

Specific TFs recruit BAF complexes to activate immune response genes in BMDMs (42). ChIP enrichment analysis (ChEA) of pathways downregulated in *Dpf2*^Δ/Δ^ macrophages showed striking enrichment in targets of NRF2 (Figure 2G). NRF2 binds and activates target genes with antiinflammatory and antioxidant cytoprotective properties (11). Hallmark gene set enrichment analysis (GSEA) confirmed that *Dpf2*^Δ/Δ^ macrophages had dysregulated expression of inflammatory response genes, ROS pathway genes, and KRAS and immune signaling pathway genes (Figure 2H), many of which are controlled by NRF2 (43). Thus, DPF2 depletion results in defective expression of cytoprotective genes that are dependent on NRF2. We also found a positive enrichment in IFN-α and IFN-γ response genes (Figure 2G) that was specific to M1 macrophages. NRF2 is required to inhibit the expression of a subset of proinflammatory cytokines (44) and IFN-regulated genes (45, 46). Therefore, DPF2 loss impairs both NRF2-activating and -repressing functions, uncovering a DPF2/NRF2 axis in the control of inflammatory gene expression in macrophages.

### DPF2 loss promotes hyperproliferation and unrestrained activation of Th cells.

**Figure 3:**
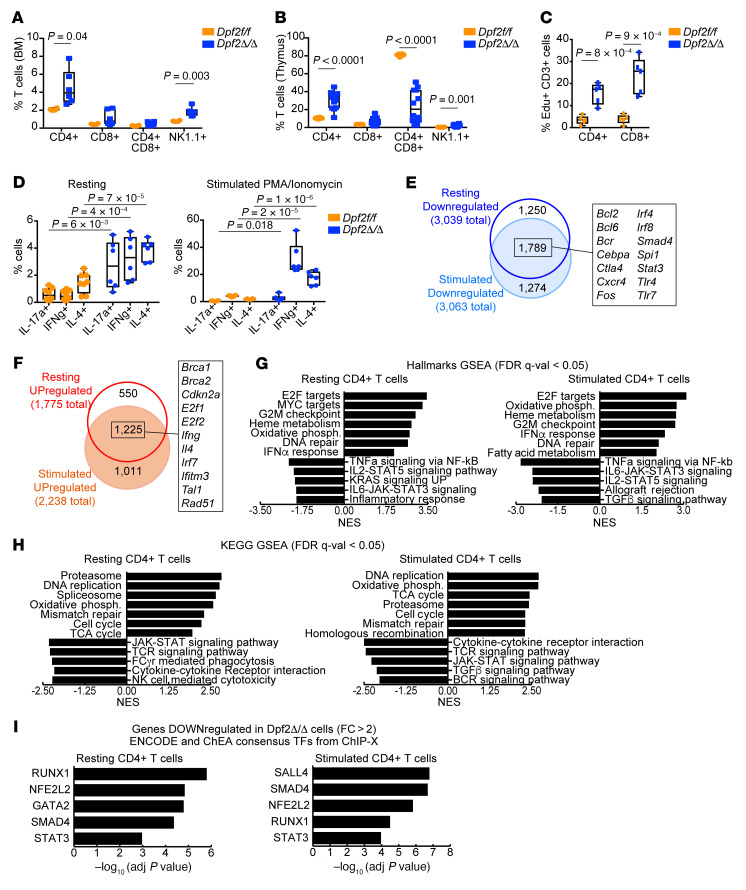
Absence of *Dpf2* leads to the expansion and increased cytokine production of T cell populations. (**A**) Frequency of CD3^+^ T and NK cells (CD3^–^NK1.1^+^) in BM of 28-day-old mice. (**B**) Same as **A**, but in the thymus. (**C**) Percentage of EdU^+^CD3^+^ splenic T cell populations. (**D**) Flow cytometric analyses of intracellular cytokines expressed from sorted CD4^+^ T cell subsets after stimulation. (**E**) Overlap between genes downregulated after *Dpf2* loss in resting or stimulated CD4^+^ T cells (*q* < 0.05, fold change >2). Highlighted are a few of the 1,789 genes downregulated after *Dpf2* loss in both conditions. (**F**) Same as in **E**, but for genes that were upregulated. (**G**) Hallmark GSEA of gene expression programs enriched in *Dpf2*^Δ/Δ^ compared with *Dpf2^fl/fl^* CD4^+^ T cells. (**H**) KEGG GSEA of pathways enriched in *Dpf2*^Δ/Δ^ CD4^+^ T cells. (**I**) ENCODE and ChEA consensus TFs from ChIP coupled with high-throughput techniques (ChIP-X) analysis obtained from genes that were downregulated (*q* < 0.05, fold change >2) in *Dpf2*^Δ/Δ^ compared with *Dpf2^fl/fl^* CD4^+^ T cells. Plots represent the mean ± SEM. *P* values were calculated using a 2-tailed, unpaired Student’s *t* test except for panel **D**, which was calculated using 2-way ANOVA. Absence of a *P* value indicates a nonsignificant difference.

T cells initiate and resolve inflammatory processes. We found increased CD4^+^ T cells and NK cells (CD3e^–^NK1.1^+^) in the BM and thymus of end-stage *Dpf2*^Δ/Δ^ mice (Figure 3, A and B). *Dpf2*^Δ/Δ^ splenic CD3^+^ T cells also showed hyperproliferation (Figure 3C). In addition, T cell maturation was impaired (Figure 3B and Supplemental Figure 3A). Under resting and stimulation (with PMA and ionomycin) conditions, *Dpf2*^Δ/Δ^ CD4^+^ T cells showed a dramatic increase in cytokine production (Th1, Th2, and Th17 cell subsets produce IFN-γ, IL-4, and IL-17, respectively) (Figure 3D and Supplemental Figure 3B). The CD3^+^, CD4^+^, and CD8^+^ T cells in the *Dpf2*^Δ/Δ^ liver and lung infiltrates were consistent with an active inflammatory response (Supplemental Figure 3, C–E). Although *Dpf2*^Δ/Δ^ Tregs showed increased FOXP3 expression in vitro, we did not find an increased in Tregs in the liver or lung infiltrates of *Dpf2*^Δ/Δ^ mice (Supplemental Figure 3, F and G).

To understand how DPF2 regulates Th cell proliferation and activation, we performed RNA-Seq in sorted splenic CD4^+^ T cells (Supplemental Table 1). Principal component analysis (PCA), as well as the overlaps between differentially expressed genes in resting and stimulated *Dpf2*^Δ/Δ^ Th cells, revealed a high similarity between the transcriptional profiles of resting and stimulated Th cells, confirming a basal state of activation induced by DPF2 loss (Supplemental Figure 3H and Figure 3, E and F). *Dpf2* deletion resulted in a larger number of downregulated genes than upregulated genes (Supplemental Figure 3I). Similar to *Dpf2*^Δ/Δ^ macrophages, genes upregulated in resting and stimulated *Dpf2*^Δ/Δ^ Th cells were enriched for E2F4 targets and cell-cycle/proliferation pathways (Supplemental Figure 3J and Figure 3, G and H); genes downregulated were enriched in immune signaling and inflammatory response pathways (Figure 3, G and H) and in NRF2 targets (Figure 3I), suggesting impaired NRF2 function. Both resting and stimulated T cells also showed positive enrichment in genes related to the IFN response (Figure 3G), a result aligned with our ex vivo flow analyses and with reports that NRF2 deficiency enhances IFN expression (47, 48). Collectively, our results provide strong evidence of multilineage proinflammatory defects driven by *Dpf2* deletion that converged on at least 2 key regulatory pathways, those regulated by E2F4 and by NRF2. The result is an aberrant state of inflammation and tissue infiltration that impairs organ function and leads to death.

### Alterations of myeloid and T cell populations in Dpf2^Δ/Δ^ mice arise postnatally.

**Figure 4:**
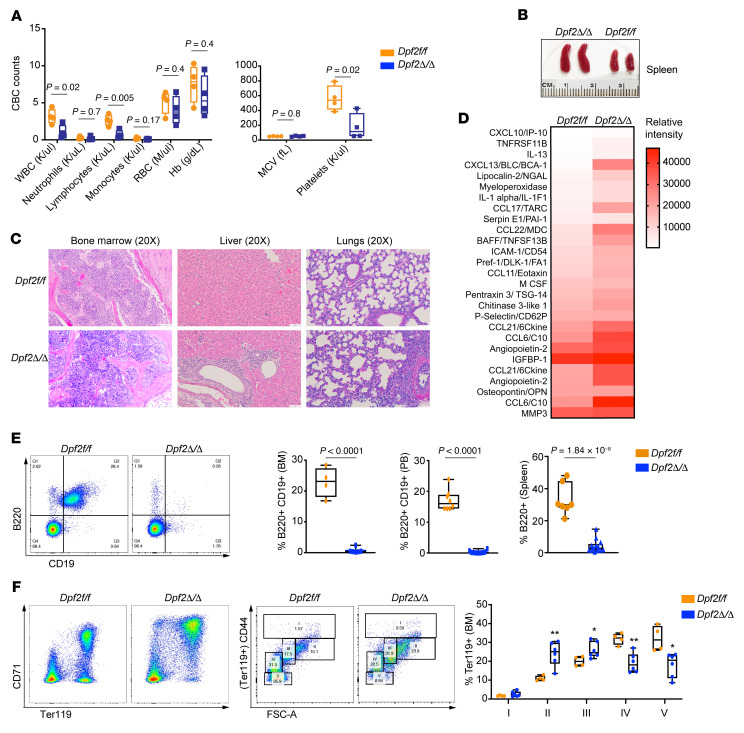
BM of *Dpf2^Δ/Δ^* mice displays early expansion of macrophages and T cells, with impaired B cell and erythroid cell differentiation. (**A**) PB CBC from 14-day-old mice (*n* = 4). MCV, mean corpuscular volume. (**B**) Representative images of spleens from 14-day-old mice. (**C**) H&E staining of BM, liver, and lungs from 14-day old mice. Scale bars: 50 μm. (**D**) Plasma cytokine levels in 14-day-old mice. Values correspond to the mean spot pixel density relative to background in 4 mice/genotype. (**E**) Representative FACS plots of BM B cells and quantification of B cell frequency in BM, PB, and spleen of end-stage *Dpf2*^Δ/Δ^ mice and *Dpf2^fl/fl^* littermates. (**F**) Representative FACS plots of BM erythroid cell maturation based on the expression of CD71 and Ter119 surface markers (107). FACS profiles resolve 5 distinct clusters: clusters IV and V (low CD44 and smaller size) correspond to orthochromatic erythroblasts, reticulocytes, and mature RBCs; clusters I, II, and III (higher CD44 and larger size) correspond to immature nucleated erythroblasts, specifically pro-erythroblasts, basophilic erythroblasts, and polychromatic erythroblasts, respectively. FSC-A, forward scatter area. Bar graph data represent the mean ± SEM. *P < 0.05 and **P < 0.01, by 2-tailed, unpaired Student’s *t* test.

To determine when the inflammatory phenotypes of *Dpf2*^Δ/Δ^ mice appear, we examined hematopoiesis in fetuses and 14-day-old mice. We did not observe gross alterations in fetal hematopoiesis (Supplemental Figure 4, A and B), but 14-day-old *Dpf2*^Δ/Δ^ mice showed pancytopenia, splenomegaly, and aberrant splenic and thymic architecture (Figure 4, A and B, and Supplemental Figure 4C). We also found histiocytic infiltrations in the liver and lungs and elevated serum levels of the same cytokines detected in end-stage mice (Figure 4, C and D), demonstrating that systemic inflammation was present early after birth.

We used mass cytometry time of flight (CyTOF) to simultaneously measure 31 cellular markers (Supplemental Table 2) on BM samples from 14-day-old mice (*n* = 3 mice/genotype). Visualization of stochastic neighbor embedding (viSNE) analyses uncovered marked differences in the abundance of 22 cell clusters between the *Dpf2*^Δ/Δ^ and *Dpf2^fl/fl^* mice (Supplemental Figure 4, D and E): seven clusters were substantially less abundant and included primarily mature erythroid and lymphoid cells; 8 clusters were more abundant and represented mostly myeloid cell/macrophage populations (CD11b^+^CD16^+^F4/80^+^), and potentially immature lymphoid cell populations (B220^+^CD27^+^CD19^+^). The frequency of mature B cells and erythroid BM cells was dramatically decreased in *Dpf2*^Δ/Δ^ mice (Supplemental Figure 4F), a result confirmed by flow cytometry (Figure 4, E–F). Fourteen-day-old *Dpf2*^Δ/Δ^ mice also showed a reduced frequency of granulocytes (Gr-1/Ly6G^+^) and a striking expansion of macrophages (CD11b^+^F4/80^+^), specific CD4^+^ T cells, NK cells (B220^+^Nkp46^+^), and conventional DCs (Supplemental Figure 4, G–I). This analysis confirmed that DPF2 loss impaired B cell and erythroid differentiation, while increasing the frequency of specific myeloid cell, T cell, and NK cell populations that reflect and contribute to unrestrained inflammation.

### DPF2-deficient hematopoietic stem and progenitor cells show increased proliferation, myeloid skewing and defective engraftment.

**Figure 5:**
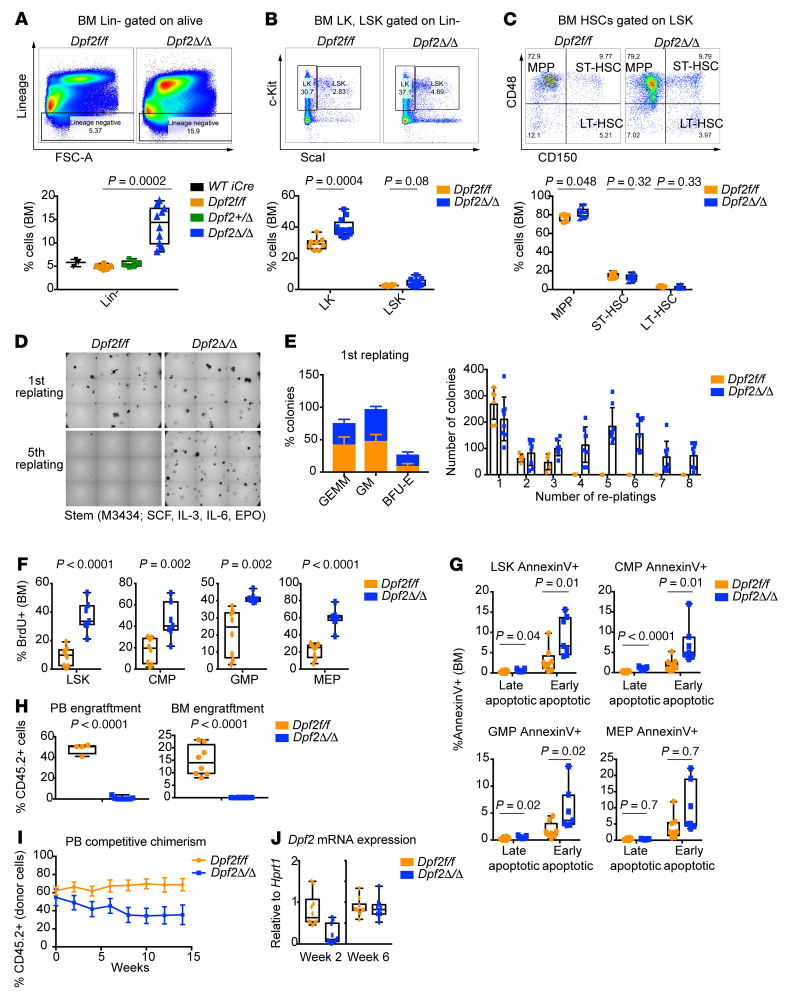
*Dpf2* deletion enhances HSC replating capacity, proliferation, and apoptosis and impairs HSC transplantability. (**A**) Representative FACS and quantification of Lin^–^ cell populations. (**B**) Same as in **A**, but for LK and LSK cell populations gated from BM Lin^–^ cells. (**C**) Representative FACS and frequency of BM MPPs (Lin^–^c-Kit^+^Sca1^+^CD48^+^CD150^–^), short-term HSCs (ST-HSCs) (Lin^–^c-Kit^+^Sca1^+^CD48^+^CD150^+^), and long-term HSCs (LT-HSCs) (Lin^–^c-Kit^+^Sca1^+^CD48^–^CD150^+^) gated on LSK cells. (**D**) Colonies on the first and fifth replatings of Lin^–^ BM cells. (**E**) Colony types on the first plating (*n* = 6) and quantification of CFU during 8 consecutive replatings. GEMM, CFU granulocyte, erythrocyte, macrophage, megakaryocyte; BFU-E, erythroid burst-forming units. (**F**) Percentage of BrdU^+^ cells within the indicated populations. (**G**) Percentage of annexin V^+^ cells within the indicated populations. Early apoptotic cells: annexin V^+^, viability dye^–^; late apoptotic and necrotic cells: annexin V^+^, viability dye^+^. (**H**) PB and BM engraftment of donor cells (CD45.2^+^) from 28-day-old mice transplanted into sublethally irradiated recipient mice. Engraftment was analyzed 4 weeks after transplantation. (**I**) Flow analyses of PB competitive chimerism. (**J**) *Dpf2* mRNA expression levels in PB, 2 and 6 weeks after tamoxifen administration. Plots represent the mean ± SEM. *P* values were calculated using a 2-tailed, unpaired Student’s *t* test except for data in panel **A**, for which a 2-way ANOVA was applied. Absence of a *P* value indicates a nonsignificant difference.

We next characterized the distinct hematopoietic stem and progenitor cells (HSPCs) using both traditional flow cytometry (on end-stage mice) and CyTOF (on 14-day-old mice). Contour plots of lineage-negative cells (Lin^–^) identified striking differences in the abundance of these immature cell populations (Supplemental Figure 5A), with an increased proportion of Lin^–^ (Figure 5A) and Lin^–^c-Kit^+^ (LK) cells (Figure 5B) in *Dpf2*^Δ/Δ^ mice. Although the frequency and number of Lin^–^c-Kit^+^ Sca1^+^ (LSK) cells was not significantly altered (Figure 5B), we observed a slight increase of multipotent progenitors (MPPs) (Figure 5C), a decrease of common myeloid progenitors (CMPs) and granulocyte-macrophage progenitors (GMPs), and an increase of megakaryocyte-erythrocyte progenitors (MEPs) (Supplemental Figure 5B). We also observed a decrease in lymphoid progenitors, and an increase in early lymphoid-committed precursors (49) (Supplemental Figure 5C). Taking BM cellularity into consideration, we found increased numbers of MPPs and MEPs and decreased numbers of CMPs, GMPs, and lymphoid progenitors in the *Dpf2*-deficient mice (Supplemental Figure 5, D and E, and data not shown). Collectively, our data indicate that *Dpf2* deficiency results in an increased frequency of HSPCs.

To investigate the basis for this increased frequency of HSPCs, we evaluated their self-renewal capacity and found that *Dpf2*^Δ/Δ^ Lin^–^ cells were able to serially replate indefinitely (Figure 5, D and E). In the first replating, *Dpf2*^Δ/Δ^ Lin^–^ cells had a higher frequency of granulocyte-macrophage (GM) CFU than did cells from *Dpf2^fl/fl^* mice (Figure 5E), suggesting an increased frequency of monocyte and neutrophil precursors. Furthermore, *Dpf2*^Δ/Δ^ BM cells failed to generate erythroid colonies (Supplemental Figure 5F) but did generate colonies that predominantly contained monocytes and macrophages (Supplemental Figure 5G). In liquid culture, DPF2 loss significantly impaired erythroid differentiation, while promoting myeloid differentiation toward macrophages (Supplemental Figure 5, H and I).

*Dpf2*^Δ/Δ^ HSCs and HSPCs showed increased proliferation as determined by in vivo BrdU and Click-iT EdU assays (Figure 5F and Supplemental Figure 5J), consistent with the increased BM Ki67 expression (Supplemental Figure 5K). However, the frequency of late and early apoptotic HSPCs was also increased in the BM of *Dpf2*^Δ/Δ^ mice (Figure 5G).

Quiescence is required for the homing and lodging of HSCs in transplantation experiments (50). In line with their loss of quiescence, *Dpf2*^Δ/Δ^ BM cells from end-stage mice showed an impaired homing capacity upon transplantation and were absent from the PB and BM of recipient mice 4 weeks after transplantation (Supplemental Figure 5L and Figure 5H). Given the homing defects of *Dpf2*^Δ/Δ^ BM cells, we generated tamoxifen-inducible *Dpf2*^Δ/Δ^ mice; as expected, *ER-Cre Dpf2^fl/fl^* and control donor cells (CD45.2^+^) engrafted equally (Figure 5I). However, tamoxifen-induced *Dpf2* deletion markedly impaired HSC competitive fitness over time (Figure 5I); by week 6 after tamoxifen administration, *Dpf2*^Δ/Δ^ cells were no longer detectable, and the remaining CD45.2^+^ *ER-Cre^+^ Dpf2^fl/fl^* cells had not achieved successful *Dpf2* deletion (Figure 5J). These data indicate that *Dpf2*^Δ/Δ^ HSCs had impaired homing and transplant capacity. Furthermore, our results suggest that the absence of DPF2 promoted a state of emergency hematopoiesis, whereby HSPCs more readily exited quiescence to produce myeloid and Th cells that contributed to a state of chronic inflammation.

### Dpf2 deletion in HSPCs prevents the activation of NRF2-dependent gene expression programs.

**Figure 6:**
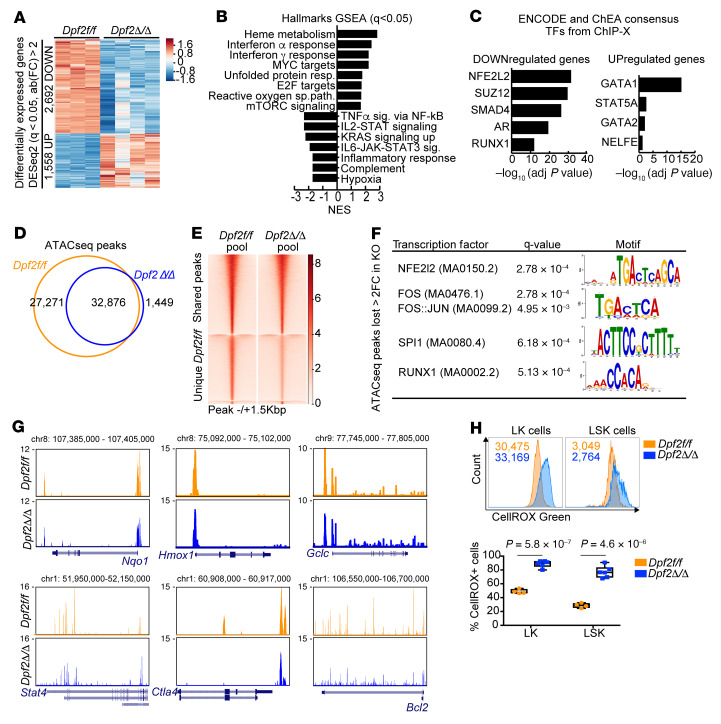
*Dpf2* deficiency in HSPCs results in the downregulation of NRF2 target genes. (**A**) Differentially expressed genes (*q* < 0.05, fold change >2) in LK cells from 28-day-old mice. (**B**) GSEA of hallmark gene expression profiles of *Dpf2*^Δ/Δ^ compared with *Dpf2^fl/fl^* LK cells. resp., response; reactive oxygen sp. path., ROS path; sig., signaling. (**C**) ENCODE and ChEA consensus TFs from ChIP-X analysis of genes deregulated in *Dpf2*^Δ/Δ^ LK cells. (**D**) Overlap between ATAC-Seq peaks called in *Dpf2^fl/fl^* and *Dpf2*^Δ/Δ^ cells. (**E**) Average ATAC-Seq signal in LK cells. Top cluster corresponds to 32,876 peaks common between control and KO cells; middle cluster corresponds to 27,271 peaks unique in *Dpf2^fl/fl^* cells; bottom cluster corresponds to 1,449 peaks unique in *Dpf2*^Δ/Δ^ cells. (**F**) TF motif analysis of ATAC-Seq peaks lost in *Dpf2*^Δ/Δ^ LK cells (i.e, with >2-fold higher signal in *Dpf2^fl/fl^* vs. *Dpf2*^Δ/Δ^ cells). Motifs were ranked on the basis of *q* value significance. FC, fold change. (**G**) UCSC Genome Browser snapshots showing pooled ATAC-Seq signals in *Dpf2^fl/fl^* and *Dpf2*^Δ/Δ^ LK cells. (**H**) Flow cytometric analysis of ROS production by BM LK and LSK cells from end-stage mice. Plots represent the mean ± SEM. *P* values were calculated using a 2-tailed, unpaired Student’s *t* test.

To identify transcriptional networks that underlie the phenotypes of *Dpf2*-null HSPCs, we performed RNA-Seq and found that 2,692 genes and 1,558 genes were significantly downregulated and upregulated, respectively, in *Dpf2*^Δ/Δ^ BM LK cells (*q* < 0.05; fold change >2; Figure 6A and Supplemental Table 1). Hallmark GSEA revealed that *Dpf2*^Δ/Δ^ HSPCs displayed transcriptional defects similar to those of *Dpf2*^Δ/Δ^ macrophages and T cells (Figure 6B, Figure 2H, and Figure 3G), including downregulation of hematopoietic cell differentiation, wound healing, and inflammatory response pathways (Figure 6B and Supplemental Figure 6A). NRF2 targets were enriched among the genes downregulated (Figure 6C), a result confirmed by interrogation of a previously published NRF2 signature (15, 51) (Supplemental Figure 6B).

Chromatin accessibility in BM LK cells showed loss of nearly half of the accessible peaks in *Dpf2*^Δ/Δ^ cells (27,271 of 60,147 peaks; Figure 6, D and E, and Supplemental Figure 6C), mostly in intronic and intergenic regions (Supplemental Figure 6D), suggesting enhancer misregulation. We focused on the 7,400 peaks that lost assay for transposase-accessible chromatin using sequencing (ATAC-Seq) signal at least 2-fold in *Dpf2*^Δ/Δ^ LK cells, primarily located at introns and intergenic regions (Supplemental Figure 6E), and closely annotated to 3,420 genes enriched in critical pathways that regulate HSC proliferation and quiescence, including Rap1 (52), PI3K-AKT (53), and MAPK and RAS (54) signaling pathways (Supplemental Figure 6F). TF Motif analyses showed enrichment in NRF2/NFE2L2 binding motifs (Figure 6F), confirming loss of accessibility and transcription at antioxidant and antiinflammatory loci such as *Nqo1*, *Hmox1*, and *Gclc* (Figure 6G). ATAC-Seq peaks gained in *Dpf2*^Δ/Δ^ LK cells were enriched in CTCF and repressor element-1–silencing transcription factor (REST) binding sites (Supplemental Figure 6G). The REST complex interacts with and requires the BAF complex to repress neuronal gene expression (55); our results suggest a similar requirement in HSPCs. Nevertheless, our transcriptomic and genome accessibility data implicate DPF2 in controlling NRF2-dependent genes and enhancers, which are centrally involved in restraining HSC quiescence and inflammatory and oxidative stress responses. In agreement with the genomics analyses, *Dpf2*^Δ/Δ^ LK and LSK cells showed a dramatic increase in ROS production (Figure 6H).

### Dpf2 deletion in HSPCs displaces BRG1 and NRF2 from enhancers, downregulating gene transcription.

**Figure 7:**
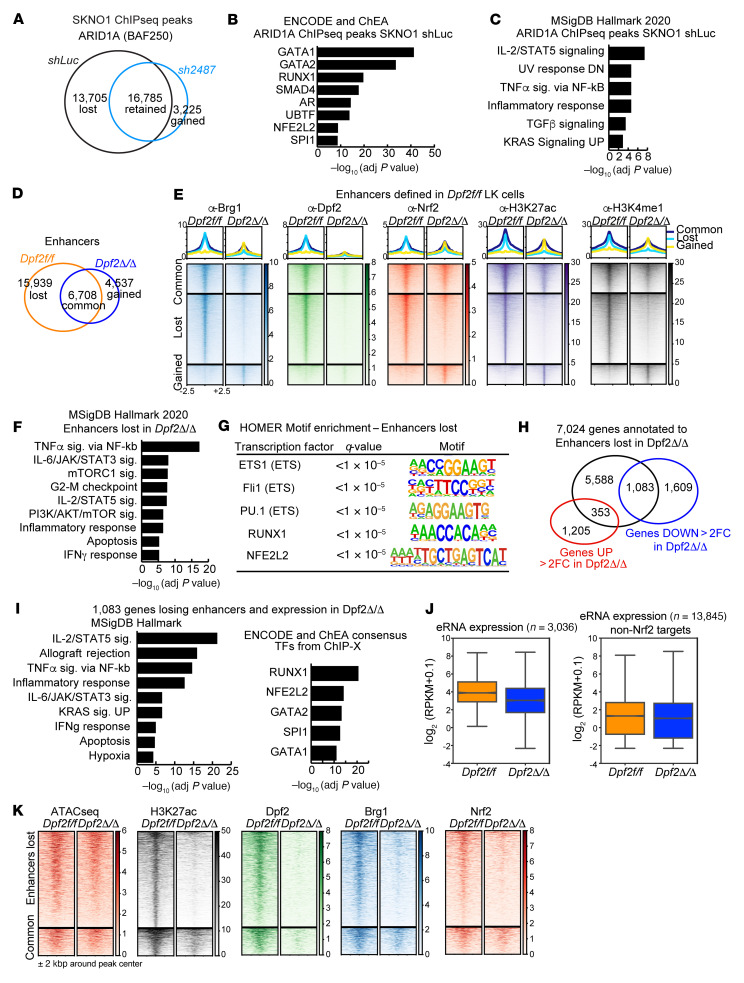
*Dpf2* deletion impairs BRG1 and NRF2 binding and activation of cognate regulatory enhancers. (**A**) Overlap between ARID1A ChIP-Seq peaks in SKNO-1 *shLuc* and *shDPF2* (hairpin number 2487) cells. (**B**) ENCODE and ChEA consensus TFs from ChIP-X analysis of genes that lose ARID1A occupancy in SKNO-1 *shDpf2* cells compared with *shLuc* cells. (**C**) Molecular Signatures Database (MSigDB) hallmark analyses of pathways enriched among genes that lose ARID1A occupancy. (**D**) Overlap between enhancers identified in *Dpf2^fl/fl^* and in *Dpf2*^Δ/Δ^ LK cells. (**E**) BRG1, DPF2, NRF2, H3K27ac, and H3K4me1 CUT&RUN signal at enhancers that are common, gained, or lost in *Dpf2*^Δ/Δ^ versus *Dpf2^fl/fl^* LK cells. (**F**) MSigDB hallmark analysis of 7,024 genes annotated to the 15,939 enhancers lost in *Dpf2*^Δ/Δ^ LK cells. (**G**) HOMER motif enrichment analysis on the 15,939 enhancers lost in *Dpf2*^Δ/Δ^ LK cells. (**H**) Overlap between the 7,024 genes annotated to enhancers lost in *Dpf2*^Δ/Δ^ LK cells, and the differentially expressed genes in *Dpf2*^Δ/Δ^ compared with *Dpf2^fl/fl^* LK cells (*q* < 0.05, fold change >2). (**I**) MSigDB hallmark analysis and ENCODE and ChEA consensus TFs from ChIP-X analysis obtained from 1,083 genes that lose nearby enhancers and are downregulated in *Dpf2*^Δ/Δ^ LK cells. (**J**) Box-and-whisker plots of eRNAs expressed from DPF2-NRF2 co-occupied active enhancers in *Dpf2^fl/fl^* and *Dpf2*^Δ/Δ^ LK cells (*n* = 3,036, left plot; ***P < 0.001, by 2-tailed Student’s *t* test) and from active enhancers not occupied by NRF2 (*n* = 13,845, right plot). RPKM, reads per kilobase per million mapped reads. Error bars represent the SD. The center line of the box plots represents the median, and the upper and lower bounds of the whiskers represent the maximum and minimum values, respectively. (**K**) ATAC-Seq, H3K27ac, DPF2, BRG1, and NRF2 signals at enhancers that are lost or maintained (“common”) in *Dpf2*^Δ/Δ^ LK cells. adj, adjusted.

To define the mechanism whereby *Dpf2* loss impairs NRF2-dependent gene expression, we generated acute myeloid leukemia (AML) cell lines that express doxycycline-inducible *Dpf2-* or *shRenilla*-directed shRNAs and performed RNA-Seq and ChIP-Seq (Supplemental Figure 7A). We used SKNO-1 cells, which have high *DPF2* expression (28). Knockdown (KD) of *DPF2* did not affect the expression of other BAF subunits (24) (Supplemental Figure 7A). Hallmark GSEA of *DPF2*-KD cells matched that of primary mouse *Dpf2*^Δ/Δ^ LK cells, with upregulation of cell-cycle and heme metabolism pathways and downregulation of inflammatory response genes (Supplemental Figure 7B). ChIP-Seq showed decreased chromatin binding of AT-rich interactive domain–containing protein 1A (ARID1A) after *DPF2* depletion (Figure 7A). Furthermore, genes annotated to the ARID1A-lost peaks showed enrichment for NRF2 targets (Figure 7, B and C). Thus, our AML cell line data suggest that *DPF2* depletion reduced the genome-wide occupancy of the canonical BAF complex on NRF2 target sites.

In mouse HSPCs, we found that the expression of BAF complex subunits was also unaffected by *Dpf2* loss (Supplemental Figure 7C and Supplemental Figure 1A). Subcellular fractionations showed that *Dpf2* deletion triggered the accumulation of NRF2 in the total and cytoplasmic fractions — consistent with the presence of a proinflammatory environment — but not in the nuclear fraction (Supplemental Figure 7D), suggesting that DPF2 deficiency impaired nuclear NRF2 accumulation and function. CUT&RUN assays in *Dpf2^fl/fl^* BM LK cells showed co-occupancy of NRF2 with BRG1, DPF2, and H3K27ac, indicative of active transcription (Supplemental Figure 7, E and F). Genes annotated to co-occupied regions were primarily related to the cell cycle, immune signaling, and oxidative stress (Supplemental Figure 7G). *Dpf2* loss resulted in a global reduction of BRG1 and H3K27ac binding, but the overall effect on NRF2 binding was limited (Supplemental Figure 7, E and F). *Dpf2* loss primarily affected the accessibility of enhancers enriched at NRF2-binding sites (Supplemental Figure 6E and Figure 6F) and led to a global reduction in H3K27ac-marked enhancers and BRG1 binding (Figure 7, D and E), suggesting an altered DPF2-BRG1 chromatin association. Remarkably, despite the limited effects on NRF2 global occupancy, binding of NRF2 was precisely lost at these enhancers (compare Supplemental Figure 7, E and F, with Figure 7E). Enhancers that lost BRG1, NRF2, and H3K27ac in *Dpf2*^Δ/Δ^ LK cells were near genes related to immune signaling, cell-cycle control and inflammatory and IFN response pathways (Figure 7F) and were enriched in NRF2 binding motifs (Figure 7G). In contrast, genes annotated to enhancers gained in *Dpf2*^Δ/Δ^ LK cells were associated with heme metabolism or IFN response pathways and enriched for GATA binding motifs (Supplemental Figure 7, H and I).

Interestingly, 40% (*n* = 1,083) of the genes downregulated more than 2-fold after *Dpf2* loss were located near the enhancers lost in *Dpf2*^Δ/Δ^ LK cells (Figure 7H). These genes were NRF2 targets and functionally enriched in inflammatory response and immune signaling pathways (Figure 7I), including well-known NRF2 targets involved in antiinflammatory and antioxidant responses (Supplemental Figure 7J). In contrast, 21% (*n* = 326) of the genes upregulated in *Dpf2*^Δ/Δ^ were near the enhancers gained after *Dpf2* loss (Supplemental Figure 7K). These 326 genes were enriched in GATA targets (Supplemental Figure 7L) and functionally related to heme metabolism and ferroptosis, a pathway upregulated in the absence of NRF2 (56).

To more directly determine whether DPF2 regulates enhancers of NRF2-dependent genes, we examined enhancer RNA (eRNA) levels at the 3,036 active enhancers in *Dpf2^fl/fl^* LK cells that are co-occupied by DPF2 and NRF2 and lost after *Dpf2* deletion (corresponding to the 15,939 enhancers shown in Figure 7D after excluding regions that overlapped with reference genes). Although these enhancers showed a statistically significant (*P* < 0.001) decrease in eRNA expression (Figure 7J), chromatin accessibility, and NRF2 occupancy (Figure 7K), we observed no difference for enhancers not co-occupied by NRF2 (in *Dpf2^fl/fl^* LK cells) (Figure 7J). Collectively, our data support a model in which loss of DPF2 displaces BRG1 and NRF2 from their cognate enhancers, leading to transcriptional downregulation of genes that modulate inflammation and oxidative stress.

### CDDO-imidazole treatment partially reverses Dpf2KO-driven phenotypes in HSPCs and mice.

**Figure 8:**
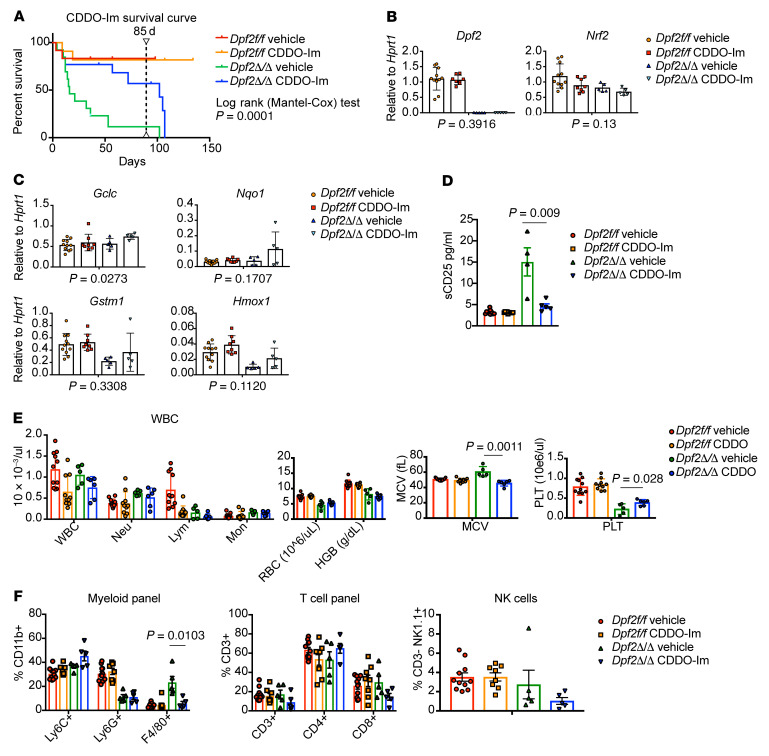
Pharmacological stimulation of NRF2 can restore gene expression and prolong survival of the *Dpf2^Δ/Δ^* mice. (**A**) Kaplan-Meier survival curves of mice treated with vehicle or CDDO-Im. Treatment was interrupted after 85 days (dashed line). A log-rank (Mantel-Cox) text was performed to determine significant differences in the survival of *Dpf2*^Δ/Δ^ mice treated with vehicle or CDDO-Im. (**B** and **C**) Expression of *Dpf2* and *Nrf2* (**B**), and NRF2 target genes (**C**) in LK cells from *Dpf2^fl/fl^* and *Dpf2*^Δ/Δ^ mice treated with vehicle or CDDO-Im. *P* value for vehicle-treated versus CDDO-Im–treated *Dpf2*^Δ/Δ^ mouse data are indicated below each plot. (**D**) sCD25 in plasma from the indicated groups of mice. (**E**) PB counts in mice treated for 2 weeks with vehicle or CDDO-Im. (**F**) Flow cytometric analyses of splenic cell populations. Data represent the mean ± SEM. *P* values were calculated using 2-way ANOVA.

To ascertain the dependency of NRF2 in the *Dpf2*^Δ/Δ^-driven phenotypes, we used the NRF2 inducer CDDO-imidazole (CDDO-Im) (57), which reduces T cell cytokine expression, promotes antioxidant gene expression, and protects against LPS-induced mortality (58). CDDO-Im was nontoxic and increased the expression of NRF2 target genes in BM *Dpf2^fl/fl^* Lin^–^ cells (Supplemental Figure 8A). CDDO-Im significantly improved the survival of *Dpf2*^Δ/Δ^ mice (Figure 8A), and interruption of treatment led to a drop in viability, confirming that the increased survival was treatment dependent (Figure 8A).

CDDO-Im treatment of BM *Dpf2^fl/fl^* cells resulted in the accumulation of NRF2 and increased expression of NRF2 target genes (Supplemental Figure 8, B and C) and impaired the enhanced self-renewal capacity of *Dpf2-*deficient HSPCs (Supplemental Figure 8D). Treatment of mice with CDDO-Im for 2 weeks did not alter *Dpf2* or *Nrf2* expression in BM LK cells (Figure 8B), but enhanced canonical NRF2 target gene expression in cells lacking *Dpf2* (Figure 8C). CDDO-Im also significantly reduced soluble CD25 (sCD25) plasma levels, improved thrombocytopenia, and decreased the frequency of splenic macrophages in *Dpf2*^Δ/Δ^ mice (Figure 8, D–F). Overall, our data favors a model in which loss of *Dpf2* impairs the nuclear accumulation of NRF2, decreasing NRF2-regulated enhancer activity and the expression of NRF2 target genes. Treatment with CDDO-Im stabilized NRF2 nuclear levels and partially rescued the expression of NRF2 target genes in *Dpf2*^Δ/Δ^ LK cells ex vivo and in vivo, improving *Dpf2*-KO–driven phenotypes and survival of the mice.

Altogether, our data show that DPF2 exerted a multilineage control of the inflammatory response that converged on NRF2, suggesting the potential of therapeutic exploitation of the functional relationship between DPF2 and NRF2 and potentially use NRF2 activators in the setting of DPF2-BAF complex functional abnormalities.

## Discussion

We have identified a requirement of the BAF complex subunit DPF2 in modulating inflammation and show that (a) DPF2 inhibits the proliferation, self-renewal, and myeloid skewing of HSPCs, (b) promotes the activation of tissue-repairing M2 macrophages, and (c) suppresses T cell activation and proliferation. Decreased NRF2 function is one of the mechanisms underlying the *Dpf2*^Δ/Δ^-driven phenotypes, as DPF2 affects NRF2-dependent gene expression via enhancer regulation. Pharmacological activation of NRF2 improved the survival and health of the *Dpf2*^Δ/Δ^ mice, and thus DPF2 and NRF2 appeared to function at the core of hematopoietic homeostasis to prevent chronic inflammation.

*Dpf2* loss leads to the hyperproliferation of HSCs, but also macrophages and T cells that drive the formation of histiocytic and inflammatory infiltrates. Consistent with this notion, we found that depletion of macrophages with chlodronate-containing liposomes prolonged the survival of *Dpf2*^Δ*/*Δ^ mice (data not shown). Our mechanistic data implicate E2F4, a TF that represses cell-cycle genes to maintain quiescence, in DPF2-dependent gene expression in macrophages and T cells. E2F4 target genes are upregulated in *Dpf2*^Δ*/*Δ^ immune effector cells, consistent with E2F4 exerting a repressive effect on cell-cycle gene expression. Further experiments will be required to define the contribution of E2F4 to the observed phenotypes.

We focused on the interplay between NRF2 and DPF2, given the phenotypic similarities of the corresponding genetically modified mice. In HSCs, *Nrf2* deficiency leads to increased proliferation and self-renewal, as well as impaired survival and homing/chimerism after transplantation (19, 21). *Nrf2^–/–^* mice also succumb prematurely to an autoimmune disease characterized by multiorgan inflammatory lesions (59), and *Nrf2-*deficient mice develop chronic inflammation (60, 61). *Nrf2^–/–^* and *Dpf2*^Δ/Δ^ mice similarly display a basal hyperinflammatory state in the absence of inflammatory stress. Although this could suggest that DPF2 may regulate immune cell differentiation and/or trafficking in homeostatic conditions, the phenotype is also consistent with the role of NRF2 in stress-free inflammation (15, 16, 62–64). *Dpf2*^Δ/Δ^ HSCs also have transcriptional and functional profiles similar to those reported for *Nrf2^–/–^* HSCs. NRF2 and BRG1 interact to regulate antioxidant gene expression (65–67). We could not detect a direct physical interaction between NRF2 and DPF2 or BRG1, but demonstrate that they co-occupied target sites and that the absence of DPF2 impaired BRG1 and NRF2 genomic association and NRF2-dependent gene expression. Our results suggest that BRG1 relocalization is likely a consequence of impaired BAF complex assembly and/or chromatin targeting. DPF2 binds acetylated and crotonylated histone residues via its tandem PHD finger domains (28, 68), and we found co-occupancy of BRG1, DPF2, and NRF2 at sites marked with H3K27ac. Histone crotonylation positively regulates transcription, and increased H3K18cr levels are found at specific inflammatory genes in response to LPS treatment in macrophages (69, 70). Although future studies are warranted to address the specific role of histone crotonylation and its contribution to the observed *Dpf2*-KO phenotypes, our data indicate that *Dpf2* deletion probably affects localization of the BAF complex to specific gene regulatory regions that are decorated with acetylated — and potentially crotonylated — histones.

Hematopoiesis-specific deletion of BAF subunits affects HSC survival and differentiation (7–10, 71–74). For example, the BAF subunit SMARCD2 is required for granulocytic differentiation, and *Smarcd2*-deficient mice develop macrophage infiltration in the lungs, without an increase in serum levels of proinflammatory cytokines, excluding the possibility of systemic inflammation (75). To our knowledge, neither *Vav1-Cre*– nor *Mx1-Cre*–mediated deletion of any BAF subunits reported thus far has resulted in the lethal systemic inflammation that we observed in *Dpf2*^Δ/Δ^ mice. Such selectivity could be due to the requirement of DPF2 for NF-κB–dependent transcriptional activation (30, 32). Our data align with these reports, as loss of DPF2 in HSPCs and immune effector cells impairs the induction of TNF-α signaling via NF-κB. NF-κB and the KEAP1/NRF2 pathways exhibit crosstalk at numerous levels, with generally opposing anti- and proinflammatory functions for NRF2 and NF-κB (60, 76–78). Future studies are warranted to elucidate the transcriptional dependencies of these factors in HSPCs and immune effector cells.

Unrestrained inflammation is an underlying driver of cancer and inflammatory and metabolic diseases. Although our models were aimed at modeling hematopoiesis-specific loss of *Dpf2* and not inflammatory conditions, the *Dpf2*-KO phenotypes are consistent with acute and chronic stress, and thus the linkage between NRF2 and DPF2 may have important clinical implications in the context of inflammatory diseases. Globally, BAF complex mutations account for more than 20% of mutations in human cancer (79), but the impact of these mutations in the development of chronic inflammation is poorly understood. NRF2 is implicated in neurodegenerative diseases, metabolic disorders, cancer, and chronic inflammatory disorders (43, 56, 80), leading to clinical efforts directed toward the modulation of NRF2 activity to promote a range of cytoprotective benefits, including redox homeostasis, dampening and resolution of inflammation, and suppression of fibrosis (81).

Our study provides compelling evidence of a multilineage functional relationship between DPF2-BAF complexes and the KEAP1/NRF2 pathway and establishes a scientific basis for therapeutic interventions in chronic inflammatory disorders, neurodegenerative diseases, and cancers driven by DPF2-BAF complex mutations.

## Methods

### Mouse models.

Global homozygous deletion of *Dpf2* is lethal in mice (82). Conditional C57BL/6 SJL *Dpf2^f/+^* mice (B6.129S6-*Dpf2^tm1.2Grc^*/J; stock no. 019144) were crossed with *Vav1-Cre* mice (B6.Cg-*Commd10^Tg(Vav1-icre)A2Kio^*/J; stock no. 008610), *Mx1Cre^+^* mice (B6;Cg-*Tg(Mx1-cre)1Cgn*/J; stock no. 003556), and UBC-Cre-ERT2 mice [B6.Cg-*Ndor1^Tg(UBC-cre/ERT2)1Ejb^*/1J] from The Jackson Laboratory to generate *Dpf2^fl/fl^*, *Dpf2*^+/Δ^, *Dpf2*^Δ/Δ^, and *Cre-*only control mice. Experiments were performed using end-stage *Dpf2*^Δ/Δ^ mice (approximately 28 days old) or 14-day-old mice with age-matched littermates. Fetal liver cells were isolated from embryos at E14.5.

For the *Mx1Cre^+^* model, 6- to 8-week-old mice were injected i.p. with poly(I:C) (InvivoGen, tlrl-pic-5) at 10 mg/kg every other day for a total of 3 doses. PB counts and flow analyses were performed 2 weeks after poly(I:C) administration. Histological analyses were performed in end-stage *Dpf2*^Δ/Δ^ mice and age-matched *Dpf2^fl/fl^* mice that received poly(I:C) at the same time.

For homing and transplantation models, a total of 5 million BM cells were isolated from *Dpf2*^Δ/Δ^ end-stage mice, *Dpf2^fl/fl^*, or *WT* *iCre* control mice (CD45.2^+^) and injected via the tail vein into sublethally (4.5 Gy) or lethally (7.5 Gy) irradiated B6.SJL mice (CD45.1^+^, The Jackson Laboratory, stock no. 002014). Dose-response curves were generated to experimentally determine the doses of irradiation using an in-house irradiator (Xstrahl RS225). PB and BM cells were analyzed by CBC and flow cytometry 20 hours (homing analysis) or 4 weeks after transplantation (engraftment).

For the competitive transplant model, 2.5 million total BM cells from 6- to 8-week-old *ERT2-Cre*, *Dpf2^fl/fl^*, and *ERT2-Cre* *Dpf2^fl/fl^* mice were transplanted together with 0.5 million helper cells into the tail veins of lethally irradiated (7.5 Gy) B6.SJL recipient mice. Two weeks later, PB samples from the recipient mice were analyzed by flow cytometry to assess engraftment. To induce *Dpf2* depletion, recipient mice were treated with tamoxifen (20 mg/kg) administered i.p. for 5 consecutive days. The percentage (mean ± SEM.) of CD45.1^+^ and CD45.2^+^ chimerism in the PB was examined, as well as *Dpf2* mRNA expression levels.

### Leukemia cell lines.

Doxycycline-inducible *shRNA-DPF2* (DPF2_1357_v1 (TGGGTATGAAATATGAAGTGGA) and DPF2_2487_v1 (TACTAATGTTTAGAATACAGGA) vectors were purchased from the RNAi/CRISPR-Cas9 core facility at Memorial Sloan Kettering Cancer Center (MSKCC). SKNO-1 cells transduced with lentiviruses were selected with 2 μg/mL puromycin for at least 7 days. Induction of hairpins was performed by adding 1 μg/mL doxycycline hyclate (Selleck Chemicals, WC2031, catalog S4163) for 7 days.

### Western blot analysis.

Cells were lysed in NETN buffer (50 mM Tris-HCl, pH 7.5; 150 mM NaCl; 1 mM EDTA; 1% NP40, phosphatase and protease inhibitor cocktail). The following antibodies were used: anti-DPF2 (Abcam, ab134942); anti-BRG1/SMARCA4 (Abcam, ab110641); anti-BAF155/SMARCC1 (Cell Signaling Technology, 11956); anti-BAF47/SNF5/SMARCB1 (Diagenode, C15410317); anti-H3 (Abcam, ab10799); anti-GAPDH (MilliporeSigma, G8795); anti-NRF2 (R&D Systems, MAB3925); anti-TRX1 (Cell Signaling Technology, 15140S); and anti-NQO1 (Cell Signaling Technology, 62262S).

### Histological analyses, IHC, and plasma analyses.

CBC was measured by an automated blood count (Hemavet System 950FS). May-Grünwald-Giemsa staining (Thermo Fisher Scientific, catalog 22-050-272) was used to stain PB smears. Histological analyses were performed as previously described (83), and samples were stained with H&E.

The following antibodies were used for IHC analysis: anti-CD68 (Leica Biosystems, PA0273); anti-CD69 (Abcam, 202909); anti-Ki67 (Cell Signaling Technology, 12202S; Leica Biosystems, PA0230) anti-galectin/MAC2 (Abcam, ab76245); anti-CD3 (Cell Signaling Technology, 99940S); anti-CD4 (Leica Biosystems, PA0427); anti-CD8 (Leica Biosystems, PA0183); and anti-FOXP3 (Abcam, ab215206). For BM and liver fibrosis analyses, a reticulin staining kit (MilliporeSigma, HT102A-1KT) was used.

For plasma analyses, PB samples were collected in heparinized tubes and centrifuged, and plasma was frozen or directly assayed. Plasma was used for the cytokine array analyses (R&D Systems, ARY028); the ferritin ELISA kit (ALPCO, 41-FERMS-E01); the sCD25 ELISA kit (G-Biosciences, IT5809); and chemistry profiling (HESKA; catalog 6330, COMP/EWRAP).

### Flow cytometric analysis and cell sorting.

Cells from mouse BM, PB, spleens, and thymi were stained as previously described (83) and analyzed using the FACSCanto II cytometer or sorted using FACSAria II cell sorter (BD). Data analysis was performed using FACSDiva, version 8.0.1 (BD Biosciences) and FlowJo, version 10.1 (TreeStar). Hematopoietic stem and lineage cell populations were stained using previously described protocols and antibodies (83).

In vivo cell-cycle analyses were performed using BrdU (APC BrdU Flow kit, BD Biosciences, 552598) and EdU staining kits (Click-iT Plus EdU Alexa Fluor 488 Flow Cytometry Assay kit, Thermo Fisher Scientific, C10632). BrdU was administered i.p. at 1 mg/g BW, and BM cells were harvested 1 hour after injection; EdU was administered i.p. at 2 mg/g BW, and organs were harvested 4 hours after injection (84). Analysis of apoptosis in vivo was performed using antibodies against PerCP–Cy5.5–annexin V and eFluor 506 (eBioscience 65-0866-14) as viability dye.

### BMDMs, phagocytosis assays, and polarization analysis.

Total BM cells from approximately 28-day-old *Dpf2^fl/fl^* and *Dpf2*^Δ/Δ^ mice were harvested, and BMDMs were obtained following a published protocol (85) and stimulated with 100 U/mL IFN-γ (Peprotech, 315-05-250 μg) and 100 ng/mL LPS-EB Ultrapure (InvivoGen, tlrl-3pelps) or 10 ng/mL IL-4 (Peprotech, 214-14) or a carrier control (PBS).

Phagocytosis assays were performed as described previously (86). As a control, day-7 BMDMs were processed in parallel, except that *E. coli* was not added.

For flow analyses, BMDMs stimulated for 48 hours were stained with M1 (F4/80 and CD80) or M2 (F4/80 and CD206) macrophage markers.

### CD4^+^ T cell activation flow analyses.

Splenic CD4^+^ cells from approximately 28-day-old mice were obtained using the CD4^+^ T cell isolation kit (Miltenyi Biotec, 130-104-454) or by sorting with the BD FACSAria II cell sorter. CD4^+^ cells were processed using the Mouse Th1/Th2/Th17 (BD Biosciences, 560758) and Th17/Treg (BD Biosciences, 560767) phenotyping kits. Cells were cultured for 3–4 hours with or without 50 ng/mL PMA and 1 μg/mL ionomycin and then harvested for RNA-Seq or flow analysis. The following antibodies were used: PerCP-Cy5.5 anti–mouse CD4 (BD Biosciences, 550954), PE anti–mouse IL-17a (BD Biosciences, 559502), FITC anti–mouse IFN-γ (BD Biosciences, 554411), APC anti–mouse IL-4 (BD Biosciences, 554436), and Alexa Fluor 647 anti–mouse FOXP3 (BD Biosciences, 560402).

### CyTOF analyses.

Total BM samples from three 14-day-old *Dpf2*^Δ/Δ^ and *Dpf2^fl/fl^* mice were cryopreserved in BAMBAKER media (Wako Chemicals), thawed, stained, and then prepared for mass cytometry as described previously (87). The antibodies used for mass cytometry are in Supplemental Table 2.

After acquisition on a Helios mass cytometer (Fluidigm), data were normalized (88) using standard metal-loaded beads, software, and procedures recommended by the manufacturer (Fluidigm). Data were arcsinh transformed, and an appropriate cofactor was set for each channel following established procedures (89, 90). A *t*-distributed stochastic neighbor embedding (*t*-SNE) analysis was performed using 29 of the measured markers on an equal number of live single cells, rhodium intercalator negative, from each of the 6 mouse BM samples (*n* = 170,340 total cells; *n* = 28,390 cells selected randomly from 6 samples) (91). The resulting common, 2D embedding of the data were analyzed in R. To identify populations of phenotypically similar cells, the FlowSOM R package (92) was used on the low-dimensional, *t*-SNE projection of the data with the target number of 22 clusters chosen on the basis of expert knowledge of the expected cell types. Marker enrichment modeling (MEM) was then used to quantitatively describe the phenotype of those cells within a given FlowSOM cluster (93–95). Positive MEM scores denote enrichment for a protein feature, and negative scores signify lack of a protein feature.

Examination of specific T cell and myeloid/DC types was performed on the Astrolabe Diagnostics platform (96), with FlowSOM clustering and labeling done using the Ek’Balam algorithm (96) with defined cell subset definitions (97, 98). Differential abundance analysis was performed using the edgeR R package (99–101).

### Clonogenic assays.

Total or Li^–^ BM cells (10,000 cells) from mice isolated using the Direct Lineage Cell Depletion kit (Miltenyi Biotec, 130-110-470) were seeded in the indicated Methocult media (STEMCELL Technologies). For Supplemental Figure 8D, cells were seeded in Methocult M3434 containing vehicle (DMSO) or 250 nM CDDO-Im. Colonies were scored on a STEMvision instrument (STEMCELL Technologies) or analyzed by flow cytometry on day 7 of culturing. Cells were replated weekly by seeding 10,000 cells/well.

### In vitro myeloid differentiation assays.

Differentiation assays were performed as described before (102). Day 0 corresponds to freshly isolated Lin^–^ cells. The following antibodies were used: anti–CD11b-FITC (BD Pharmingen, 557396); anti–Ly6C-PE-Cy7 (BD Pharmingen, 560593); anti–Ly6G-APC (BD Biosciences, 560599); and anti–F4/80-PE (Invitrogen, Thermo Fisher Scientific, 12-4801-80). eFluor 450 (eBioscience, 65-0863-18) was used as the viability dye.

### RNA-Seq of LK cells.

RNA from approximately 200,000 BM LK cells from approximately 1-month-old *Dpf2^fl/fl^* and *Dpf2*^Δ/Δ^ mice was extracted using the RNEasy Plus Micro kit (QIAGEN, 74034). rRNA was removed using the NEBNext rRNA Depletion kit (New England BioLabs [NEB], E6310), RNA-Seq libraries were prepared using the Illumina TruSeq Total-Stranded RNA-Seq prep kit (NuGEN Technologies) and sequenced on an Illumina NextSeq 500 High-Output kit platform (paired-end, 75 bp reads) to obtain more than 40 million paired-end reads per sample. Analyses were performed as described in a previous publication (83). DESeq2 results are included in Supplemental Table 1.

For intergenic eRNA identification, we followed a previously described approach (103).

### ATAC-Seq of LK cells.

LK cells (250,000 cells) were obtained from 1-month-old *Dpf2*^Δ/Δ^ and *Dpf2^fl/fl^* mice and sorted, cryopreserved, and processed following the OMIM-ATAC-Seq protocol (104) with minor modifications (83). OMIM-ATAC-Seq libraries were amplified for 6 cycles (105). Sequencing was performed on the Illumina NextSeq 500 (75 bp paired-end reads) to obtain more than 40 million reads per sample.

ATAC-Seq chromatin-accessible regions were determined using Encyclopedia of DNA Elements (ENCODE) pipeline standards (https://github.com/ENCODE-DCC/atac-seq-pipeline; commit ID: 2b693ab).

### ChIP-Seq and CUT&RUN.

ChIP-Seq of 10 million SKNO-1 cells carrying *shLuciferase* (*shLuc*) or *shDPF2* no. 2487 was performed following a previously published protocol (106). Rabbit anti-ARID1A (BAF250A, D2A8U, Cell Signaling Technology, catalog 12354, lot no. 1; RRID: AB_2637010) was used to immunoprecipitate ARID1A. ChIP-Seq libraries were generated using the NEBNext Ultra II DNA library prep kit for Illumina (NEB, E7370L) and sequenced on the NextSeq 500 platform (single-end, 75 bp). Analysis was performed as described previously (83). Motif analysis was performed using MEME-ChIP, version 4.12.0, and then the JASPAR 2018 motif database.

For CUT&RUN experiments, BM LK cells pooled from 3–6 approximately 28-day-old *Dpf2^fl/fl^* and *Dpf2*^Δ/Δ^ mice were mildly crosslinked (1 min, 1% formaldehyde) using the Cutana ChIC/CUT&RUN Kit (EpiCypher, 14-1048). Approximately 0.5 million cells and 1 μg antibody were used per IP. The following antibodies were used: anti-BRG1 (Bethyl, A300-813A, lot no. 4); anti-DPF2 (MilliporeSigma, SAB4502621, lot no. 3111434); anti-NRF2 (R&D Biosystems, AF3925, lot no. WID0121081); anti-IgG (Diagenode, C15410206, lot no. RIG001); anti-H3K27ac (Diagenode, C15410196, lot no. A1723-0041D); and anti-H3K4me1 (Diagenode, C15410194, lot no. A1862D). Libraries were generated using the NEBNext Ultra II DNA library Prep kit for Illumina (NEB, E7370L) and sequenced on an Illumina NOVASeq 6000 (paired-end, 75 bp). Pair-end fastq files were processed with the ENCODE Transcription Factor and Histone ChIP-Seq processing pipeline (https://github.com/ENCODE-DCC/chip-seq-pipeline2). Reads were trimmed using Cutadapt, version 2.5, and aligned to the mm10 genome using Bowtie2, version 2.3.4.3. SAMtools, version 1.9, was used to convert the output file to the BAM format. Duplicates were removed using Picard Tools, version 2.20.7. Peak calling was performed with MACS2, version 2.2.4. Hypergeometric Optimization of Motif EnRichment (HOMER), version 4.11, was used for peak annotation and motif analysis. Bedtools, version 2.29.0, intersect was used to determine peak overlaps and assign target genes. Enhancers were defined by intersecting H3K27ac and H3K4me1 peak regions by at least 1 bp.

### CellROX assays.

BM Lin^–^ cells from approximately 28-day-old mice were cultured overnight in DMEM supplemented with 10 ng/mL stem cell factor (SCF) and 100 ng/mL thrombopoietin (TPO) and then processed using the CellROX Green Flow Cytometry Assay kit (Thermo Fisher Scientific, C10492). Flow cytometric analyses were performed to analyze the production of CellROX Green in LSK cells using SytoxBlue as the viability dye (Thermo Fisher Scientific, S34857).

### CDDO-Im experiments.

For the survival experiment, CDDO-Im (Fisher Scientific, 47-371-0) was dissolved freshly in vehicle (10% DMSO 10% Kolliphor in PBS) and administered to 2-week-old mice via oral gavage at 20 μmol/kg BW, 3 times per week, for up to 85 weeks. Treatment was interrupted on day 85, and survival was monitored until the death of all *Dpf2*^Δ/Δ^ mice. Mice were treated for 2 weeks to test the effects of CDDO-Im on NRF2 target gene expression, sCD25 plasma levels, and PB cell composition.

### Statistics.

All bar graph data are expressed as the mean ± SD. Statistically significant differences and *P* values were calculated using a 2-tailed, unpaired Student’s *t* test for comparisons between 2 groups, or ANOVA for comparisons between more than 2 groups. A *P* value of less than 0.05 was considered significant. Analyses were performed using GraphPad Prism, version 8 (GraphPad Software).

### Study approval.

All mice were maintained in the University of Miami animal facility under virus antibody–free (VAF) conditions. Both male and female mice were used, and no mouse was excluded in the experiments. All animal studies were approved by the IACUC of the University of Miami.

### Data availability.

RNA-Seq, ChIP-Seq, ATAC-Seq, and CUT&RUN data sets generated for this study are available in the NCBI’s Gene Expression Omnibus (GEO) database (GEO GSE192779).

## Author contributions

GM and SDN conceived the project. GM designed the studies with help from NM, and both performed most of the experiments. DK, FB, FT, and CKC performed genomics analyses. YN performed CUT&RUN experiments. CMC, CC, and SD provided experimental support. HI, FV, and SCK provided advice and pathological diagnoses. AKM, KK, and AMV carried out cytokine array experiments, IHC experiments, and ChIP-Seq in AML cell lines, respectively. RS supervised the bioinformatic analyses. CK, LM, and DB provided resources and intellectual support. GM wrote the manuscript with help from NM and SDN. SDN supervised the work.

## Supplementary Material

Supplemental data

Supplemental table 1

Supplemental table 2

## Version 1 05/18/2023

In-Press Preview

## Version 2 07/03/2023

Electronic publication

## Version 3 02/15/2024

Ramin Shiekhattar added to author list.
