# Supplementary material for: The SWI/SNF chromatin-remodeling subunit DPF2 facilitates NRF2-dependent antiinflammatory and antioxidant gene expression
Source: J Clin Invest. 2023 Jul 3;133(13):e158419. doi: 10.1172/JCI158419 (PMC10313367; doi:10.1172/JCI158419)
Supplement: Supplemental data [file jci-133-158419-s009.pdf]

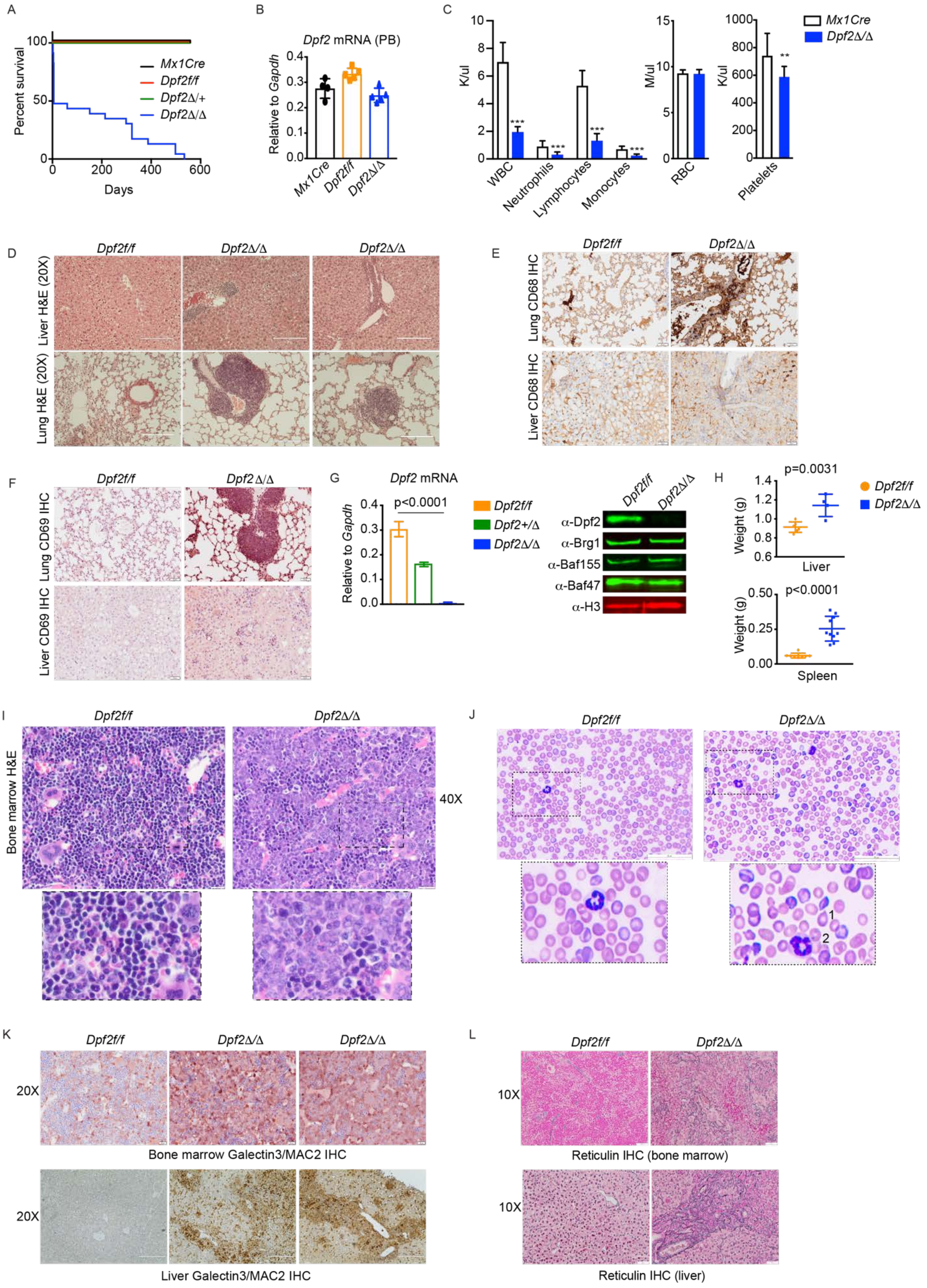
A
Percent survival
Days
Mx1Cre
Dpf2f/f
Dpf2Δ/+
Dpf2Δ/Δ
B
Dpf2 mRNA (PB)
Relative to Gapdh
Mx1Cre
Dpf2f/f
Dpf2Δ/Δ
C
K/ul
WBC
Neutrophils
Lymphocytes
Monocytes
M/ul
RBC
K/ul
Platelets
Mx1Cre
Dpf2Δ/Δ
D
Dpf2f/f
Dpf2Δ/Δ
Dpf2Δ/Δ
Liver H&E (20X)
Lung H&E (20X)
E
Dpf2f/f
Dpf2Δ/Δ
Lung CD68 IHC
Liver CD68 IHC
F
Dpf2f/f
Dpf2Δ/Δ
Lung CD69 IHC
Liver CD69 IHC
G
Dpf2 mRNA
Relative to Gapdh
p<0.0001
Dpf2f/f
Dpf2+/Δ
Dpf2Δ/Δ
Dpf2f/f
Dpf2Δ/Δ
α-Dpf2
α-Brg1
α-Baf155
α-Baf47
α-H3
H
p=0.0031
Weight (g)
Liver
p<0.0001
Weight (g)
Spleen
Dpf2f/f
Dpf2Δ/Δ
I
Dpf2f/f
Dpf2Δ/Δ
Bone marrow H&E
40X
J
Dpf2f/f
Dpf2Δ/Δ
1
2
K
Dpf2f/f
Dpf2Δ/Δ
Dpf2Δ/Δ
20X
Bone marrow Galectin3/MAC2 IHC
20X
Liver Galectin3/MAC2 IHC
L
Dpf2f/f
Dpf2Δ/Δ
10X
Reticulin IHC (bone marrow)
10X
Reticulin IHC (liver)

Supplementary Figure 1. Inflammatory infiltrates and impaired survival in *Dpf2*$^{\Delta/\Delta}$ mice

(A) Kaplan-Meier survival curves of the indicated groups of *Mx1-Cre* derived mice after Poly(I:C) administration (N=12-20 per genotype). **(B)** Relative *Dpf2* mRNA expression in PB, 2 weeks after Poly(I:C) administration. **(C)** PB CBC, 2 weeks after Poly(I:C) administration (N = 8). White blood cells (WBC), red blood cells (RBC). **(D)** Representative H&E images showing histiocytic infiltrations in liver and lung of mice analyzed 300 days after treatment with Poly (I:C). Scale bars represent 200 µm (20X). **(E)** Representative CD68 IHC images of lung and liver, 300 days after Poly (I:C). **(F)** Same as (E), for CD69 IHC. Scale bars represent 50 µm. **(G)** *Dpf2* mRNA expression levels in PB from *Vav1-Cre* crossed, 28 days old wild-type (*Dpf2*$^{f/f}$), heterozygous (*Dpf2*$^{+/\Delta}$) and Dpf2 knock-out (*Dpf2*$^{\Delta/\Delta}$) mice. Western blot analysis of the indicated BAF subunits in BM Lin- cells. **(H)** Liver and spleen weight of 28-day-old mice. **(I)** Representative images of BM H&E staining. Scale bars represent 50 µm (40X). **(J)** Wright-Giemsa stains of representative PB smears. Numbers indicate abnormal shape or size (anisocyte; 1), and Howell-Jolly bodies (2). Scale bars correspond to 50 µm. **(K)** Galectin3/MAC2 IHC staining of BM and liver sections. Scale bars represent 20 µm (for BM) and 200 µm (for liver). **(L)** Reticulin staining of BM and liver sections. Scale bars represent 50 µm (10X). All bar graph data represent mean ± s.e.m.. *P*-values were calculated using two-tail unpaired Student's t-tests, except in Figure S1G where *p*-values were calculated using ordinary one-way ANOVA. Absence of *p*-value indicates no significant difference.

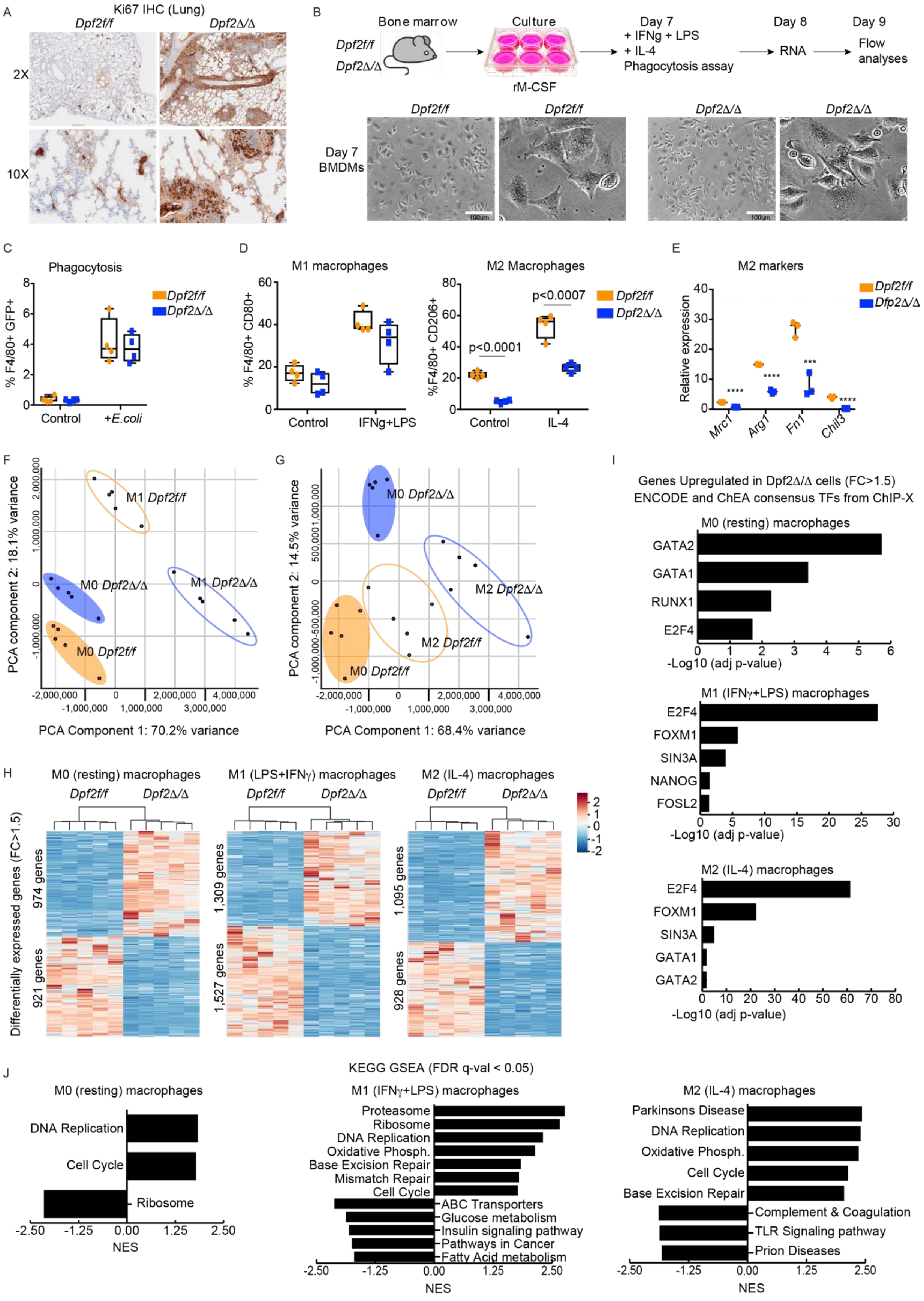
A
Ki67 IHC (Lung)
Dpf2f/f
Dpf2Δ/Δ
2X
10X
B
Bone marrow
Culture
rM-CSF
Day 7
+ IFNg + LPS
+ IL-4
Phagocytosis assay
Day 8
RNA
Day 9
Flow analyses
Day 7 BMDMs
C
Phagocytosis
% F4/80+ GFP+
Control
+E.coli
D
M1 macrophages
% F4/80+ CD80+
IFNg+LPS
M2 Macrophages
%F4/80+ CD206+
p<0.0001
p<0.0007
IL-4
E
M2 markers
Relative expression
Dfp2Δ/Δ
Mrc1
Arg1
Fn1
Chil3
F
PCA component 2: 18.1% variance
PCA Component 1: 70.2% variance
M1 Dpf2f/f
M0 Dpf2Δ/Δ
M1 Dpf2Δ/Δ
M0 Dpf2f/f
G
PCA component 2: 14.5% variance
PCA Component 1: 68.4% variance
M2 Dpf2Δ/Δ
M2 Dpf2f/f
I
Genes Upregulated in Dpf2Δ/Δ cells (FC>1.5)
ENCODE and ChEA consensus TFs from ChIP-X
M0 (resting) macrophages
GATA2
GATA1
RUNX1
E2F4
-Log10 (adj p-value)
M1 (IFNγ+LPS) macrophages
FOXM1
SIN3A
NANOG
FOSL2
M2 (IL-4) macrophages
H
Differentially expressed genes (FC>1.5)
M0 (resting) macrophages
M1 (LPS+IFNγ) macrophages
M2 (IL-4) macrophages
974 genes
921 genes
1,309 genes
1,527 genes
1,095 genes
928 genes
J
KEGG GSEA (FDR q-val < 0.05)
DNA Replication
Cell Cycle
Ribosome
NES
Proteasome
Oxidative Phosph.
Base Excision Repair
Mismatch Repair
ABC Transporters
Glucose metabolism
Insulin signaling pathway
Pathways in Cancer
Fatty Acid metabolism
Parkinsons Disease
Complement & Coagulation
TLR Signaling pathway
Prion Diseases

Supplementary Figure 2. *Dpf2*$^{\Delta/\Delta}$ macrophages show major transcriptional, proliferative and metabolic alterations associated with impaired immune activation.

(A) Representative images of Ki67 IHC staining of lung sections, taken at 2X and 10X. **(B)** Experimental strategy to obtain BMDMs, and representative pictures of day 7 BMDMs. Scale bars represent 100 µm. **(C)** Phagocytosis analysis of day 7 BMDMs treated with PBS (control) or GFP+ *E.coli*. **(D)** Frequency of BMDMs (F4/80+) untreated (control) or treated with IFNγ plus LPS (M1 macrophages; CD80+) or with IL-4 (M2 macrophages; CD206+) for 48 hours. **(E)** qRT-PCR analysis of M2-specific markers in BMDMs treated for 24 hours with IL-4. **(F)** Principal Component Analysis (PCA) of RNA-Seq datasets from resting (M0) and M1 BMDMs, based on normalized expression counts. **(G)** Same as (F), for resting (M0) and M2 BMDMs. **(H)** Heatmaps of differentially expressed genes (q<0.05; fold change>1.5) between *Dpf2*$^{\Delta/\Delta}$ and *Dpf2*$^{f/f}$ BMDMs (N=5; 28 days old) in resting conditions (M0), or after 24 hours of treatment. **(I)** ENCODE and ChEA consensus TFs from ChIP-X analysis obtained from the genes upregulated (q<0.05; fold change>1.5) in *Dpf2*$^{\Delta/\Delta}$ compared to *Dpf2*$^{f/f}$ BMDMs in the indicated conditions. **(J)** GSEA KEGG analysis of gene expression programs enriched in *Dpf2*$^{\Delta/\Delta}$ BMDMs in resting conditions (M0) or treated for 24 hours. All bar graph data represent mean ± s.e.m. P values were calculated using two-tail unpaired Student's t-tests.

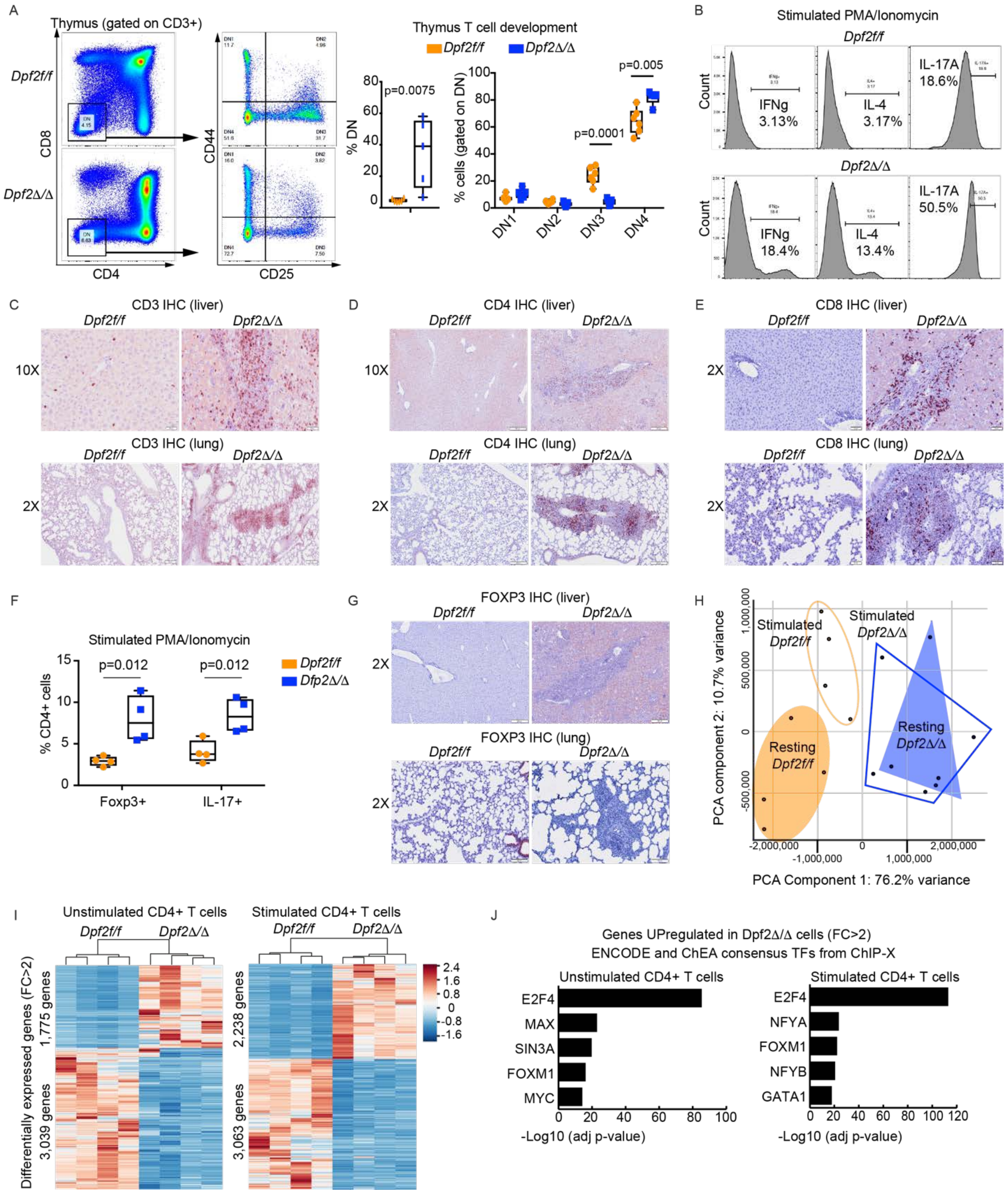

Supplementary Figure 3. *Dpf2*$^{\Delta/\Delta}$ T cells are found in inflammatory lesions and display altered gene expression consistent with hyperproliferation and activation.

(A) Representative FACS of thymic CD3+ T cell populations and analysis of Thymic T cell development. DN, Double Negative. **(B)** FACS profiles of splenic sorted CD4+ T cell subsets stimulated with PMA/Ionomycin for 5 hours. Th1, Th2 and Th17 selectively secrete IFNγ, IL-4 and IL-17a, respectively. **(C-E)** Representative images of CD3+ (C), CD4+ (D) and CD8+ (E) IHC staining of liver and lung infiltrations. Scale bars represent 50 μm (for CD3 and CD8) and 200 μm (for CD4). **(F)** Flow cytometry analysis of intracellular cytokines expressed by sorted CD4+ T cell subsets after ex vivo treatment with PMA and Ionomycin for 5 hours. Treg express FOXP3 and Th17 express IL-17. **(G)** Representative images of FOXP3 IHC staining in liver and lung. Scale bars represent 200 μm. **(H)** PCA of normalized expression counts from RNA-Seq datasets obtained from resting and stimulated CD4+ T cells. **(I)** Heatmaps of differentially expressed genes (q<0.05; fold change>2) between $Dpf2^{\Delta/\Delta}$ and $Dpf2^{f/f}$ splenic CD4+ T cells (N=4; 28 days old mice) in resting or stimulated conditions. **(J)** ENCODE and ChEA consensus transcription factors from ChIP-X analysis obtained from the genes upregulated (q<0.05; fold change>2) in $Dpf2^{\Delta/\Delta}$ splenic CD4+ T cells in the indicated conditions. All bar graph data represent mean ± s.e.m. *P* values were calculated using two-tail unpaired Student's t-tests.

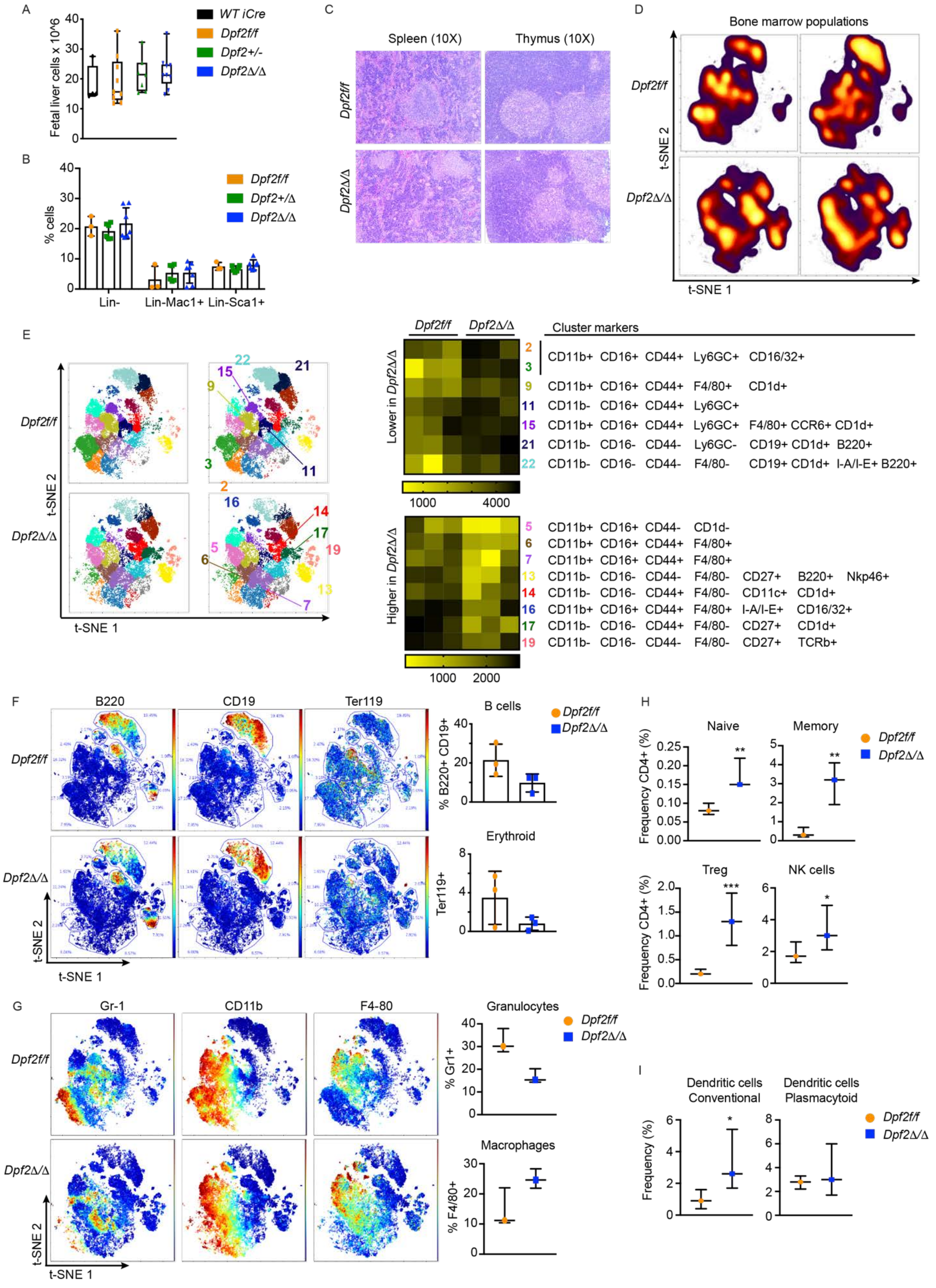
A
Fetal liver cells x 10^6
WT iCre
Dpf2f/f
Dpf2+/-
Dpf2Δ/Δ
B
% cells
Dpf2f/f
Dpf2+/Δ
Dpf2Δ/Δ
Lin-
Lin-Mac1+
Lin-Sca1+
C
Spleen (10X)
Thymus (10X)
Dpf2f/f
Dpf2Δ/Δ
D
Bone marrow populations
Dpf2f/f
Dpf2Δ/Δ
t-SNE 2
t-SNE 1
E
Dpf2f/f
Dpf2Δ/Δ
t-SNE 2
t-SNE 1
Dpf2f/f
Dpf2Δ/Δ
Cluster markers
Lower in Dpf2Δ/Δ
2
3
CD11b+ CD16+ CD44+ Ly6GC+ CD16/32+
9 CD11b+ CD16+ CD44+ F4/80+ CD1d+
11 CD11b- CD16+ CD44+ Ly6GC+
15 CD11b+ CD16+ CD44+ Ly6GC+ F4/80+ CCR6+ CD1d+
21 CD11b- CD16- CD44- Ly6GC- CD19+ CD1d+ B220+
22 CD11b- CD16- CD44- F4/80- CD19+ CD1d+ I-A/I-E+ B220+
1000
4000
Higher in Dpf2Δ/Δ
5 CD11b+ CD16+ CD44- CD1d-
6 CD11b+ CD16+ CD44+ F4/80+
7 CD11b+ CD16+ CD44+ F4/80+
13 CD11b- CD16- CD44- F4/80- CD27+ B220+ Nkp46+
14 CD11b- CD16- CD44+ F4/80- CD11c+ CD1d+
16 CD11b+ CD16+ CD44+ F4/80+ I-A/I-E+ CD16/32+
17 CD11b- CD16- CD44+ F4/80- CD27+ CD1d+
19 CD11b- CD16- CD44- F4/80- CD27+ TCRb+
1000
2000
F
B220
CD19
Ter119
Dpf2f/f
Dpf2Δ/Δ
t-SNE 2
t-SNE 1
B cells
% B220+ CD19+
Dpf2f/f
Dpf2Δ/Δ
Erythroid
Ter119+
G
Gr-1
CD11b
F4-80
Dpf2f/f
Dpf2Δ/Δ
t-SNE 2
t-SNE 1
Granulocytes
% Gr1+
Dpf2f/f
Dpf2Δ/Δ
Macrophages
% F4/80+
H
Naive
Memory
Frequency CD4+ (%)
Dpf2f/f
Dpf2Δ/Δ
Treg
NK cells
Frequency CD4+ (%)
I
Dendritic cells Conventional
Dendritic cells Plasmacytoid
Frequency (%)
Dpf2f/f
Dpf2Δ/Δ

Supplementary Figure 4. Early expansion of immature myeloid and T cell populations in *Dpf2*$^{Δ/Δ}$ mice.

(A) Absolute number of fetal liver cells in embryos of the indicated genotypes. **(B)** Frequency of the indicated fetal liver cell populations. **(C)** Representative H&E images of spleen and thymus of 14 days-old mice. Scale bars represent 50 μm (10X). **(D)** viSNE analysis on all single, Ir+ Rh- cells from BM samples (28,390 events/sample) obtained from 14-day old *Dpf2*$^{f/f}$ (top) and *Dpf2*$^{Δ/Δ}$ (bottom) mice using all markers except Rho, c-CASP3, FOXP3. Cells were arranged along X and Y axes based on the similarity of their 29-dimensional phenotypes (Supplementary Table 2). Shaded contour plots depict cellular abundance ranging from purple (low) to yellow (high). **(E)** On the same viSNE axes as in (D), identification of cell clusters by the FlowSOM algorithm, using equal sampling on all single, Ir+ Rh- cells (28,390 events/sample). Each dot represents a cell. Numbers point to specific cell clusters that are downregulated (top) or upregulated (bottom) in *Dpf2*$^{Δ/Δ}$ mice compared to *Dpf2*$^{f/f}$. Heatmaps show log2 ratios of event counts by the minimum value between all 3 samples using X-axis channel(s). Markers of each cell cluster are indicated on the right. **(F)** On the same viSNE axes, expression levels of B cell markers (B220; CD19) and the erythroid marker Ter119 in all cells that are positive for each marker. Plots show the frequency of cells in three control *Dpf2*$^{f/f}$ and three knock-out *Dpf2*$^{Δ/Δ}$ samples. **(G)** Same as (F), for the myeloid cell markers Gr-1, CD11b and F4-80 and quantification of the frequency of granulocytes (Gr1+ cells) and Macrophages (F4/80+). **(H)** Frequency of the indicated T cell subtypes and NK cells in 14-days old *Dpf2*$^{f/f}$ and *Dpf2*$^{Δ/Δ}$ BM mass cytometry samples. CD4+ Naïve T cells correspond to T helper cells (CD4+ CD3e+ CD62L+); CD4+ Memory T cells correspond to CD4+ CD3e+ CD44+ CD62L-; CD4+ T reg cells correspond to CD4+ CD3e+ FOXP3+ and NK cells correspond to CD3e- NK1.1+ cells. **(I)** Frequency of conventional (CD11b+, CD11c+) and plasmacytoid (CD11b-, B220+ CD3e- CD19- NK1.1-) dendritic cells. All bar graph data represent mean ± s.e.m. *P* values were calculated using two-tail unpaired Student's t-tests.

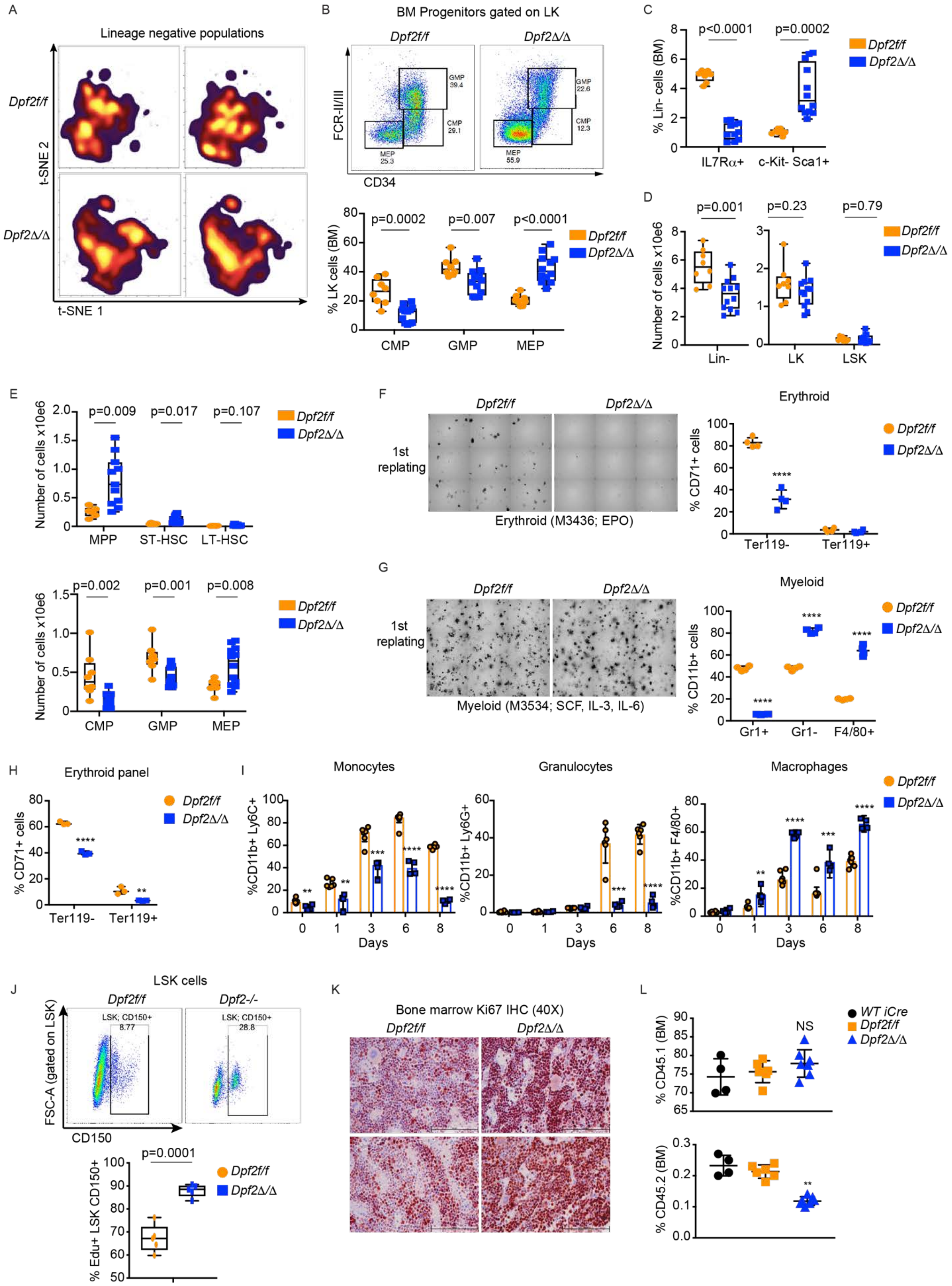
A
Lineage negative populations
Dpf2f/f
Dpf2Δ/Δ
t-SNE 2
t-SNE 1
B
BM Progenitors gated on LK
Dpf2f/f
Dpf2Δ/Δ
FCR-II/III
CD34
GMP 39.4
CMP 29.1
MEP 25.3
GMP 22.6
CMP 12.3
MEP 55.9
% LK cells (BM)
p=0.0002
p=0.007
p<0.0001
CMP
GMP
MEP
C
% Lin- cells (BM)
p<0.0001
p=0.0002
IL7Rα+
c-Kit- Sca1+
D
Number of cells x10e6
p=0.001
p=0.23
p=0.79
Lin-
LK
LSK
E
Number of cells x10e6
p=0.009
p=0.017
p=0.107
MPP
ST-HSC
LT-HSC
p=0.002
p=0.001
p=0.008
CMP
GMP
MEP
F
1st replating
Erythroid (M3436; EPO)
Erythroid
% CD71+ cells
Ter119-
Ter119+
G
Myeloid (M3534; SCF, IL-3, IL-6)
Myeloid
% CD11b+ cells
Gr1+
Gr1-
F4/80+
H
Erythroid panel
% CD71+ cells
Ter119-
Ter119+
I
Monocytes
Granulocytes
Macrophages
%CD11b+ Ly6C+
%CD11b+ Ly6G+
%CD11b+ F4/80+
Days
J
LSK cells
Dpf2f/f
Dpf2-/-
LSK; CD150+ 8.77
LSK; CD150+ 28.8
FSC-A (gated on LSK)
CD150
% Edu+ LSK CD150+
p=0.0001
K
Bone marrow Ki67 IHC (40X)
L
WT iCre
Dpf2f/f
Dpf2Δ/Δ
NS
% CD45.1 (BM)
% CD45.2 (BM)

Supplementary Figure 5. *Dpf2*$^{\Delta/\Delta}$ HSCs have myeloid skewing and impaired homing and engraftment.

(A) Contour plots of abundance of Lin- cells (CD4- CD8- CD19- B220- TCR- Nk- Ter119-). **(B)** Representative FACS and quantification of the frequency (gated on LK cells) of progenitor populations. CMP, common myeloid progenitors (Lin- c-Kit+ Sca1- CD34+ CD16/32-); GMP, granulocyte-macrophage progenitors (Lin- c-Kit+ Sca1- CD34+ CD16/32+); MEP, megakaryocyte-erythrocyte progenitors (Lin- c-Kit+ Sca1- CD34- CD16/32-). **(C)** Frequency of CLP (Common lymphoid progenitors; Lin- IL7rα+; gated on Lin-) and lymphoid precursors (Lin- c-Kit- Sca1+). **(D)** Absolute numbers of Lin- cells, Lin- c-Kit+ (LK) and Lin- c-Kit+ Sca1+ (LSK) in BM of end-stage *Dpf2*$^{\Delta/\Delta}$ and age-matched *Dpf2*$^{f/f}$ mice. **(E)** Absolute numbers of BM MPP, ST-HSC, LT-HSC and progenitor populations. **(F)** Representative image of colonies obtained from Lin- cells after 7 days of culture in Methocult M3436. Colonies were harvested and the frequency of cells with the indicated erythroid markers was analyzed. **(G)** Same as (F), for cells cultured in granulocyte-macrophage progenitor Methocult media M3534. The frequency of cells stained with myeloid surface markers Ly6G (Gr-1), CD11b (Mac1) and Ly71(F4/80) was analyzed. **(H)** Frequency of cells with the indicated erythroid surface markers after 7 days of culturing BM Lin- cells isolated in liquid media. **(I)** Frequency of myeloid (CD11b+) populations collected at the indicated days following a myeloid differentiation protocol (Methods). Cells at day 0 correspond to freshly isolated BM Lin- cells. **(J)** Representative FACS and frequency of EdU+ LT-HSCs (Lin- c-Kit+ Sca1+ CD150+). **(K)** Representative images of Ki67 IHC staining of BM sections. Scale bars represent 100 µm. **(L)** Homing analyses of donor (CD45.2+) BM cells transplanted into sub-lethally irradiated recipient mice (CD45.1+) and analyzed 20 hours after transplant. All bar graph data represent mean ± s.e.m. *P* values were calculated using two-tail unpaired Student's t-tests.

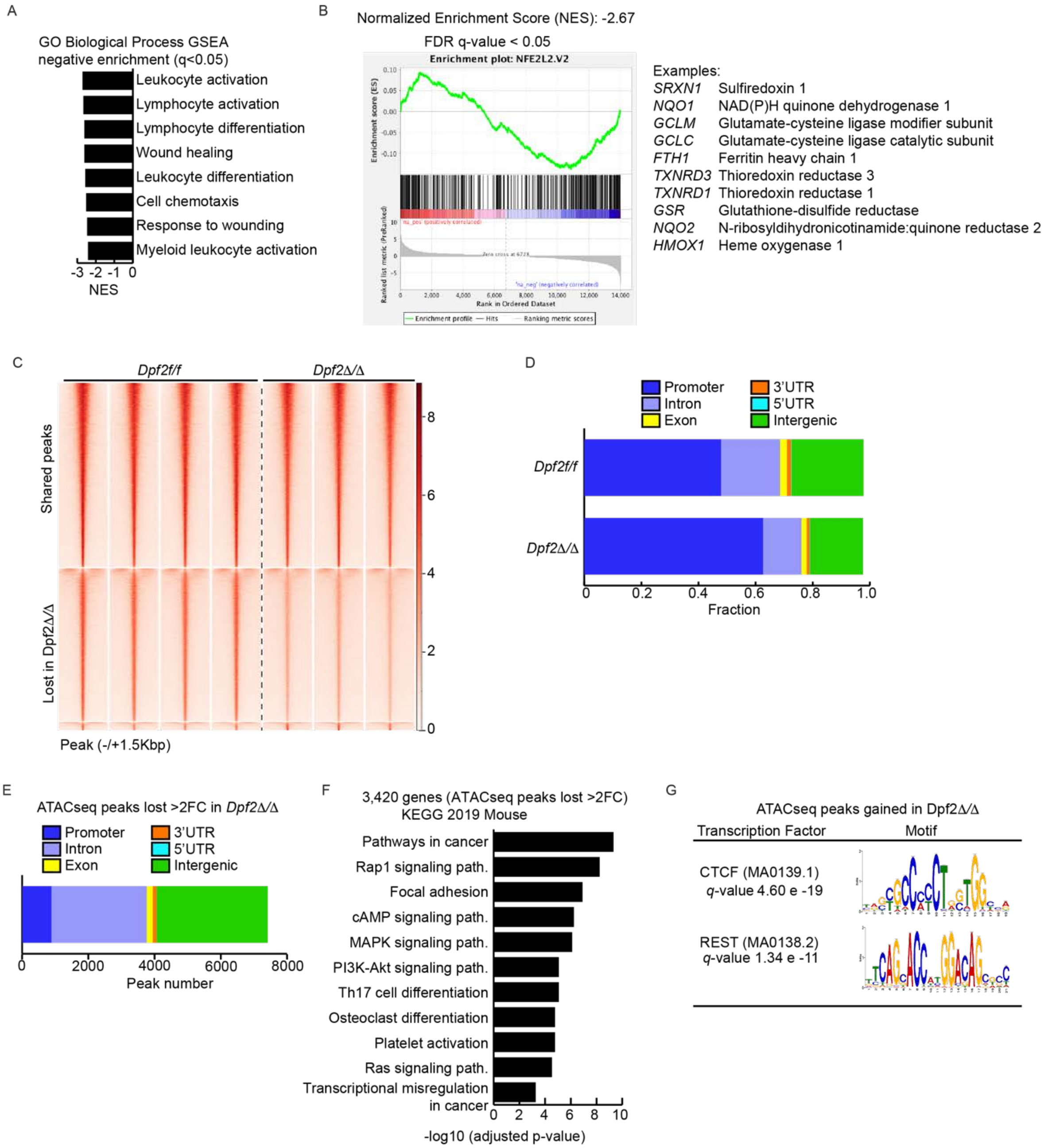
A
GO Biological Process GSEA
negative enrichment (q<0.05)
Leukocyte activation
Lymphocyte activation
Lymphocyte differentiation
Wound healing
Leukocyte differentiation
Cell chemotaxis
Response to wounding
Myeloid leukocyte activation
-3 -2 -1 0
NES
B
Normalized Enrichment Score (NES): -2.67
FDR q-value < 0.05
Enrichment plot: NFE2L2.V2
Enrichment score (ES)
Ranked list metric (PreRanked)
Rank in Ordered Dataset
Enrichment profile — Hits — Ranking metric scores
Examples:
SRXN1 Sulfiredoxin 1
NQO1 NAD(P)H quinone dehydrogenase 1
GCLM Glutamate-cysteine ligase modifier subunit
GCLC Glutamate-cysteine ligase catalytic subunit
FTH1 Ferritin heavy chain 1
TXNRD3 Thioredoxin reductase 3
TXNRD1 Thioredoxin reductase 1
GSR Glutathione-disulfide reductase
NQO2 N-ribosyldihydronicotinamide:quinone reductase 2
HMOX1 Heme oxygenase 1
C
Dpf2f/f
Dpf2Δ/Δ
Shared peaks
Lost in Dpf2Δ/Δ
8
6
4
2
0
Peak (-/+1.5Kbp)
D
Promoter
Intron
Exon
3'UTR
5'UTR
Intergenic
Dpf2f/f
Dpf2Δ/Δ
0 0.2 0.4 0.6 0.8 1.0
Fraction
E
ATACseq peaks lost >2FC in Dpf2Δ/Δ
Promoter
Intron
Exon
3'UTR
5'UTR
Intergenic
0 2000 4000 6000 8000
Peak number
F
3,420 genes (ATACseq peaks lost >2FC)
KEGG 2019 Mouse
Pathways in cancer
Rap1 signaling path.
Focal adhesion
cAMP signaling path.
MAPK signaling path.
PI3K-Akt signaling path.
Th17 cell differentiation
Osteoclast differentiation
Platelet activation
Ras signaling path.
Transcriptional misregulation in cancer
0 2 4 6 8 10
-log10 (adjusted p-value)
G
ATACseq peaks gained in Dpf2Δ/Δ
Transcription Factor
Motif
CTCF (MA0139.1)
q-value 4.60 e -19
REST (MA0138.2)
q-value 1.34 e -11

Supplementary Figure 6. *Dpf2* loss in HSPCs impairs the expression and accessibility of genes controlling differentiation and immune signaling.

(A) GSEA GO Biological Process analysis showing the gene expression pathways deregulated in *Dpf2*$^{\Delta/\Delta}$ LK cells compared to *Dpf2*$^{f/f}$ cells. **(B)** GSEA analysis of genes deregulated in *Dpf2*-deficient LK cells. Relevant examples of genes downregulated in *Dpf2*$^{\Delta/\Delta}$ LK cells are listed. **(C)** ATAC-Seq peaks (-/+ 1.5Kbp from peak center) in LK cells from independent mice. **(D)** Genomic distribution of peaks called in *Dpf2*$^{f/f}$ and *Dpf2*$^{\Delta/\Delta}$ LK cells. **(E)** Genomic distribution of peaks with at least 2-fold decrease in ATAC-Seq signal in *Dpf2*$^{\Delta/\Delta}$ LK cells compared to *Dpf2*$^{f/f}$ cells. **(F)** KEGG pathway analyses of 3,420 genes annotated to peaks that lose ATAC-Seq signal intensity at least 2-fold in *Dpf2*$^{\Delta/\Delta}$ LK cells compared to *Dpf2*$^{f/f}$ cells. **(G)** TF motif analysis on ATAC-Seq peaks gained in *Dpf2*$^{\Delta/\Delta}$ LK cells.

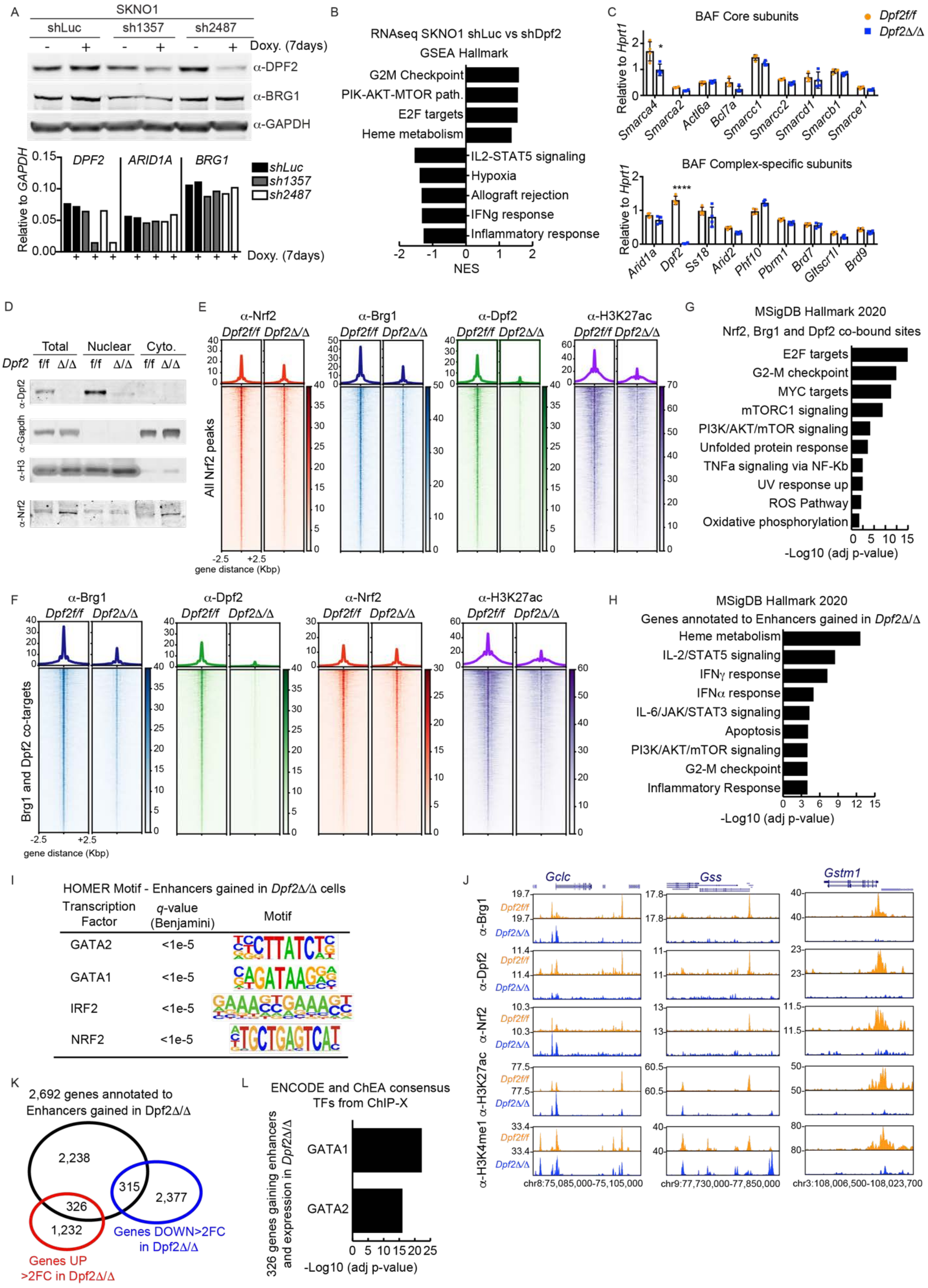
A
SKNO1
shLuc
sh1357
sh2487
Doxy. (7days)
α-DPF2
α-BRG1
α-GAPDH
Relative to GAPDH
DPF2
ARID1A
BRG1
shLuc
sh1357
sh2487
B
RNAseq SKNO1 shLuc vs shDpf2
GSEA Hallmark
G2M Checkpoint
PIK-AKT-MTOR path.
E2F targets
Heme metabolism
IL2-STAT5 signaling
Hypoxia
Allograft rejection
IFNg response
Inflammatory response
NES
C
BAF Core subunits
Dpf2f/f
Dpf2Δ/Δ
Relative to Hprt1
Smarca4
Smarca2
Actl6a
Bcl7a
Smarcc1
Smarcc2
Smarcd1
Smarcb1
Smarce1
BAF Complex-specific subunits
Arid1a
Dpf2
Ss18
Arid2
Phf10
Pbrm1
Brd7
Gltscr1l
Brd9
D
Total
Nuclear
Cyto.
α-Dpf2
α-Gapdh
α-H3
α-Nrf2
E
α-Nrf2
α-Brg1
α-Dpf2
α-H3K27ac
All Nrf2 peaks
gene distance (Kbp)
G
MSigDB Hallmark 2020
Nrf2, Brg1 and Dpf2 co-bound sites
E2F targets
G2-M checkpoint
MYC targets
mTORC1 signaling
PI3K/AKT/mTOR signaling
Unfolded protein response
TNFa signaling via NF-Kb
UV response up
ROS Pathway
Oxidative phosphorylation
-Log10 (adj p-value)
F
Brg1 and Dpf2 co-targets
H
MSigDB Hallmark 2020
Genes annotated to Enhancers gained in Dpf2Δ/Δ
Heme metabolism
IL-2/STAT5 signaling
IFNγ response
IFNα response
IL-6/JAK/STAT3 signaling
Apoptosis
PI3K/AKT/mTOR signaling
G2-M checkpoint
Inflammatory Response
I
HOMER Motif - Enhancers gained in Dpf2Δ/Δ cells
Transcription Factor | q-value (Benjamini) | Motif
GATA2 | <1e-5
GATA1 | <1e-5
IRF2 | <1e-5
NRF2 | <1e-5
J
Gclc
Gss
Gstm1
α-Brg1
α-Dpf2
α-Nrf2
α-H3K27ac
α-H3K4me1
chr8:75,085,000-75,105,000
chr9:77,730,000-77,850,000
chr3:108,006,500-108,023,700
K
2,692 genes annotated to Enhancers gained in Dpf2Δ/Δ
2,238
315
326
2,377
1,232
Genes UP >2FC in Dpf2Δ/Δ
Genes DOWN>2FC in Dpf2Δ/Δ
L
ENCODE and ChEA consensus TFs from ChIP-X
326 genes gaining enhancers and expression in Dpf2Δ/Δ
GATA1
GATA2
-Log10 (adj p-value)

Supplementary Figure 7. NRF2, DPF2 and BRG1 co-occupy regulatory regions of genes whose expression is altered following *Dpf2* loss.

(A) Western blot and qRT-PCR showing the expression of *DPF2* and the indicated BAF subunits in SKNO-1 shLuciferase and two independent shRNAs against *DPF2*, induced with doxycycline for 7 days. **(B)** GSEA Hallmark analysis of differentially expressed genes in SKNO-1 *shDPF2* cells compared to shLuciferase control. **(C)** qRT-PCR analysis of the indicated BAF subunits in BM LK cells from 28 days-old mice. Expression is calculated relative to *Hprt1*. **(D)** Subcellular fractionation of Lin- cells isolated from *Dpf2*$^{f/f}$ or *Dpf2*$^{\Delta/\Delta}$ mice. **(E)** Heatmaps of the signal of NRF2, BRG1, DPF2 and H3K27ac at all NRF2 target peaks. **(F)** Heatmaps of the signal of BRG1, DPF2, NRF2 and H3K27ac at BRG1-DPF2 co-bound sites. **(G)** MSigDB Hallmark analyses of 2,812 genes annotated to NRF2, BRG1 and DPF2 co-bound sites. **(H)** MSigDB Hallmark analysis of the 2,692 genes annotated to the 4,527 enhancers gained in *Dpf2*$^{\Delta/\Delta}$ LK cells. **(I)** HOMER motif enrichment analysis on the 4,527 enhancers gained in *Dpf2*$^{\Delta/\Delta}$ LK cells. **(J)** UCSC Genome browser examples of shared NRF2, DPF2 and BRG1 target genes with significantly decreased expression in *Dpf2*$^{\Delta/\Delta}$ LK cells. **(K)** Overlap between 2,692 genes annotated to the 4,527 enhancers gained in *Dpf2*$^{\Delta/\Delta}$ LK cells, and the differentially expressed genes in *Dpf2*$^{\Delta/\Delta}$ compared to *Dpf2*$^{f/f}$ LK cells (q<0.05; fold change>2). **(L)** ENCODE and ChEA consensus TFs from ChIP-X analysis of the 326 genes that gain enhancers and become upregulated in *Dpf2*$^{\Delta/\Delta}$ LK cells. All bar graph data represent mean ± s.e.m. *P* values were calculated using two-tail unpaired Student's t-tests.

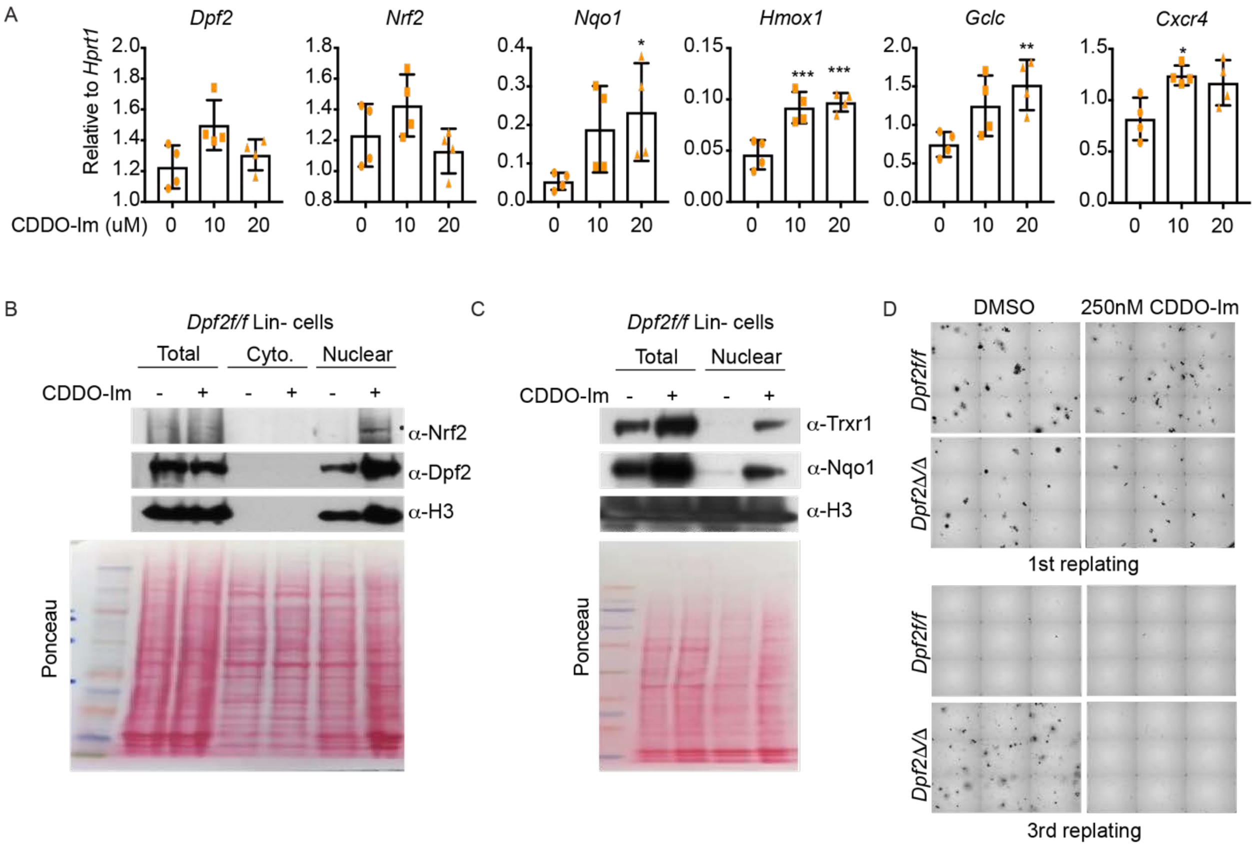

Supplementary Figure 8. Ex vivo treatment with CDDO-Im increases NRF2-target gene expression and impairs the enhanced self-renewal of DPF2-deficient HSPCs.

(A) qRT-PCR analysis of genes in BM LK cells isolated from *Dpf2*$^{f/f}$ mice treated with vehicle or CDDO-Im for 3 weeks. **(B)** Subcellular fractionation of Lin- cells isolated from 1-month old *Dpf2*$^{f/f}$ mice, treated with vehicle (-) or 300 nM CDDO-Im (+) overnight. Total, cytoplasmic (Cyto.) and nuclear extracts were run, and western blots were probed with the indicated antibodies. Ponceau staining shows comparable total protein levels. **(C)** Subcellular fractionation of *Dpf2*$^{f/f}$ Lin- cells treated with vehicle (-) or 300nM CDDO-Im (+) overnight. **(D)** Representative pictures of CFU assays on the 1st or 3rd re-plating in vehicle (DMSO) or 250 nM CDDO-Im conditions. All bar graph data represent mean ± s.e.m. *P* values were calculated using one-way ANOVA.
